# H1N1pdm09 Adjuvanted Vaccination in HIV-Infected Adults: A Randomized Trial of Two Single versus Two Double Doses

**DOI:** 10.1371/journal.pone.0039310

**Published:** 2012-06-25

**Authors:** Marilia Santini-Oliveira, Luiz A. B. Camacho, Thiago M. L. Souza, Paula M. Luz, Mauricio T. L. Vasconcellos, Carmem B. W. Giacoia-Gripp, Mariza G. Morgado, Estevão P. Nunes, Alberto S. Lemos, Ana C. G. Ferreira, Ronaldo I. Moreira, Valdiléa G. Veloso, Marilda M. Siqueira, Beatriz Grinsztejn

**Affiliations:** 1 Instituto de Pesquisa Clínica Evandro Chagas, Fundação Oswaldo Cruz, Rio de Janeiro, Rio de Janeiro, Brazil; 2 Escola Nacional de Saúde Pública, Fundação Oswaldo Cruz, Rio de Janeiro, Rio de Janeiro, Brazil; 3 Laboratório de Viroses Respiratórias, NIC-WHO, Instituto Oswaldo Cruz, Fundação Oswaldo Cruz, Rio de Janeiro, Rio de Janeiro, Brazil; 4 Laboratório de AIDS e Imunologia Molecular, Instituto Oswaldo Cruz, Fundação Oswaldo Cruz, Rio de Janeiro, Rio de Janeiro, Brazil; Istituto Superiore di Sanitá, Italy

## Abstract

**Background:**

Since human immunodeficiency virus (HIV)-infected individuals are at increased risk of severe disease from pandemic influenza A (H1N1pdm09), vaccination was recommended as a prevention strategy. The aim of the present study was to evaluate the safety, immunogenicity and persistence of the immune response after vaccination against pandemic influenza A (H1N1pdm09) with an adjuvanted vaccine in human immunodeficiency virus (HIV)-infected adults using two single and two double doses.

**Methodology/Principal Findings:**

Open label, randomized trial to evaluate the immune response following H1N1pdm09 vaccination in HIV-infected participants compared to HIV-negative controls (NCT01155037). HIV-infected participants were randomized to receive 2 single (3.75 µg hemagglutinin) or 2 double (7.5 µg hemagglutinin) doses of the vaccine, 21 days apart. Controls received one dose of the vaccine. The primary endpoint was seroconversion as measured by hemagglutination inhibition assay. Two hundred fifty six HIV-infected participants (129 and 127 randomized to single and double doses, respectively) and 71 HIV-negative controls were enrolled. Among HIV-infected participants, seroconversion increased from 46.7% and 51.7% after the first dose to 77.2% and 83.8% after the second dose of the vaccine using single and double doses, respectively. Participants aged >40 years showed higher seroconversion compared to younger participants. Seroconversion among HIV-infected women and those with nadir CD4<200 cells/mm^3^ was significantly higher with double doses. Persistence of protective antibodies six months after vaccination was achieved by 80% and 89.9% of the HIV-infected participants who received single and double doses, respectively.

**Conclusions/Significance:**

Our results support the recommendation of two double doses of adjuvanted H1N1pdm09 vaccine for HIV-infected individuals, particularly women, and those aged >40 years or with nadir CD4<200 cells/mm^3^, to achieve antibody levels that are both higher and more sustained.

**Trial Registration:**

ClinicalTrials.gov NCT01155037

## Introduction

Human immunodeficiency virus (HIV)-infected individuals are at increased risk of severe disease from numerous infections, including recurrent respiratory infections [1]. Although overall mortality associated with pandemic influenza A/H1N1 virus (H1N1pdm09) infection was considered low, individuals at risk of complications were identified, such as immunocompromised individuals that included HIV-infected [2], [3]. As a prevention strategy, vaccination was recommended to HIV-infected individuals following the same recommendations for healthy individuals [4], [5]. However, there was uncertainty regarding the best vaccination schedule for patients with impaired immunity [6], [7]. Although few studies have explored modified vaccination regimens for this population, further studies are needed to evaluate vaccine dosage and number of applications, and novel adjuvants [8].

Brazil was seriously affected by H1N1pdm09 with 34,506 influenza-like severe acute respiratory infection cases (5,747 were laboratory-confirmed cases), most occurring during the winter season of 2009 [9]. The aim of this study was to measure seroconversion after 2 single versus 2 double doses of an adjuvanted H1N1pdm09 vaccine in HIV-infected participants compared to HIV-negative controls (NCT01155037). In addition, persistence of antibody levels was evaluated for a follow-up of six months.

## Methods

The protocol for this trial and supporting CONSORT checklist are available as supporting information; see Checklist S1 and Protocol S1.

### 1. Study Design

This was an open label, randomized, phase-II trial to compare the safety, immunogenicity, and persistence of the immune response following H1N1pdm09 vaccination in HIV-infected patients randomized to receive 2 single (3.75 µg hemagglutinin) or 2 double (7.5 µg hemagglutinin) doses of the adjuvanted vaccine, 21 days apart. HIV-infected patients were also compared to non-randomized controls receiving 1 single dose. The primary endpoint of the study was seroconversion defined as serum titer ≤1∶8 before and ≥1∶32 after vaccination or baseline titer >1∶8 and at least 4-fold increase after vaccination, as measured by hemagglutination inhibition assay (HAI, described below) [10]. Seroprotection was defined by serum titer ≥1∶32.

### 2. Study Participants

HIV-infected patients who received care at the HIV/AIDS Clinic of the Evandro Chagas Clinical Research Institute, FIOCRUZ, were approached for study participation. Controls had a negative HIV rapid test result at the screening visit. Patients and controls were enrolled simultaneously from March through August 2010. Estimated sample size was defined as the higher value obtained from the two different criteria: (1) a non-inferiority limit of −10% seroconversion comparing the two groups of HIV-infected patients, and, (2) a 10 percentage point difference in seroconversion when comparing to HIV-negative controls (assuming 90% seroconversion). The study protocol was approved by the Research Ethics Committee from Instituto de Pesquisa Clínica Evandro Chagas and was registered with the Clinical Trials network (NCT01155037). All participants provided written informed consent.

Inclusion criteria for all participants were age 18–59 years. The main exclusion criteria were: receipt of another investigational vaccine or drug in the past 4 weeks, seasonal influenza vaccination in the previous 3 months, previous anaphylaxis or serious reactions to vaccines, or hypersensitivity to egg and chicken protein or ovalbumin, neurological illness, acute illness on the day of enrolment, pregnancy and breastfeeding.

### 3. Vaccine, Group Allocation and Follow-up Observation

Split, inactivated influenza virus, containing 3.75 µg of antigen equivalent to A/California/7/2009 (H1N1) v-like strain (X-179A) hemagglutinin with AS03 adjuvant (GSK) was used. Patients received two single (3.75 µg of antigen each) or two double doses (7.5 µg of antigen each), according to the randomization scheme.

Study subjects were randomized, by means of computer generated random numbers (Function *ranuni*, SAS Version 9.1.4), which had been previously assigned to one of the vaccination schemes by the statistician. Randomization used permutation blocks of size 10 with a 1∶1 allocation ratio, stratified by CD4+ count (<200 cells and ≥200 cells). The protocol was amended early in field work to discontinue stratification by CD4+ count as there were very few eligible participants with less than 200 cells per mm^3^. The assignment was printed and placed in opaque envelopes, sealed and sequentially numbered, and the whole process was concealed from the study team. After signing the consent form the next envelope in the sequence was opened and the vaccination scheme disclosed to the study team and participants. Each vial of vaccine was used in only one participant.

Blood samples were collected at baseline, 21 and 42 days, and 6 months after baseline.

Demographic and clinical data were retrieved from medical records. HIV-1 RNA viral load (Versant HIV-1 RNA 3.0 Assay, Siemens H. Diagnostics, IL, USA) and CD4 cell counts (BD Biosciences, CA, USA) were measured at baseline, 21 and 42 days after baseline.

Surveillance for influenza-like illness (ILI) started from study enrollment and continued for a follow up of 6 months. Adverse events were assessed at the clinic 1-hour after vaccination, by phone contact 20–36 hours after vaccination and in person 7-days after vaccination.

### 4. Hemagglutination Inhibition Assay (HAI)

We followed the WHO-recommended protocol to perform HAI assays [11], [12]. In brief, serum samples were treated with receptor-destroying enzyme (Denka-Seiken, Japan) and incubated with four hemagglutination units of H1N1pdm09 for 1 hour. After, guinea pig red blood cells were added to the well at final concentration of 0.5% and incubated for 1 hour, when the HAI was read. Results were expressed as the reciprocal of the highest dilution that inhibited hemagglutination. HAI titers <1∶8 were considered to have a value of 1∶4 for calculation purposes [10].

### 5. Respiratory Sample Collection and Respiratory Virus Diagnosis

Nasopharyngeal Dacron-swab specimens were collected from participants displaying acute respiratory infection and placed onto transport medium (Hanks solution with 100 U/mL penicillin and 100 µg/mL streptomycin) [13]. The RNA from clinical samples was extracted (QIAmp Viral RNA mini kit, Qiagen, Germany) and tested for the presence of seasonal and H1N1pdm09 by the WHO/CDC-recommended rRT-PCR [14]. Real time RT-PCR was performed to detect the following agents: subtypes of Influenza A, Influenza B, coronavirus (229, 43 e 63), parainfluenza (1, 2, 3 and 4), metapneumovirus, parechovirus, rhinovirus, RSV A/B, bocavirus, adenovirus and enterovirus (Fast-Track diagnosis, Luxembourg, Luxembourg).

### 6. Statistical Analysis

Statistical analyses were performed with SAS 9.1.3 (Cary, NC, USA: SAS Institute Inc., 2004) and SPSS Statistics 19 (Release Version 19.0.0; SPSS, Inc., 2010). Differences (and 95% confidence limits) between the proportions and the logarithm of antibody titers for double doses and single doses were calculated [15]. The non-inferiority limit for seroconversion and ratios of geometric mean titers were −10% and 0.5, respectively. Statistical significance of differences between proportions was assessed using the chi-squared test. Comparison of means was based on the *t*-test. Cumulative curves of antibody titers were compared across groups using the Log-rank test. All tests used 95% confidence limits. Logistic regression was used to adjust the degree of association of the vaccination schemes to seroconversion at 42 days after baseline for demographic and clinical factors. Likewise, the association of antibody titers to relevant covariates was explored in multiple regression models.

## Results

**Figure 1:**
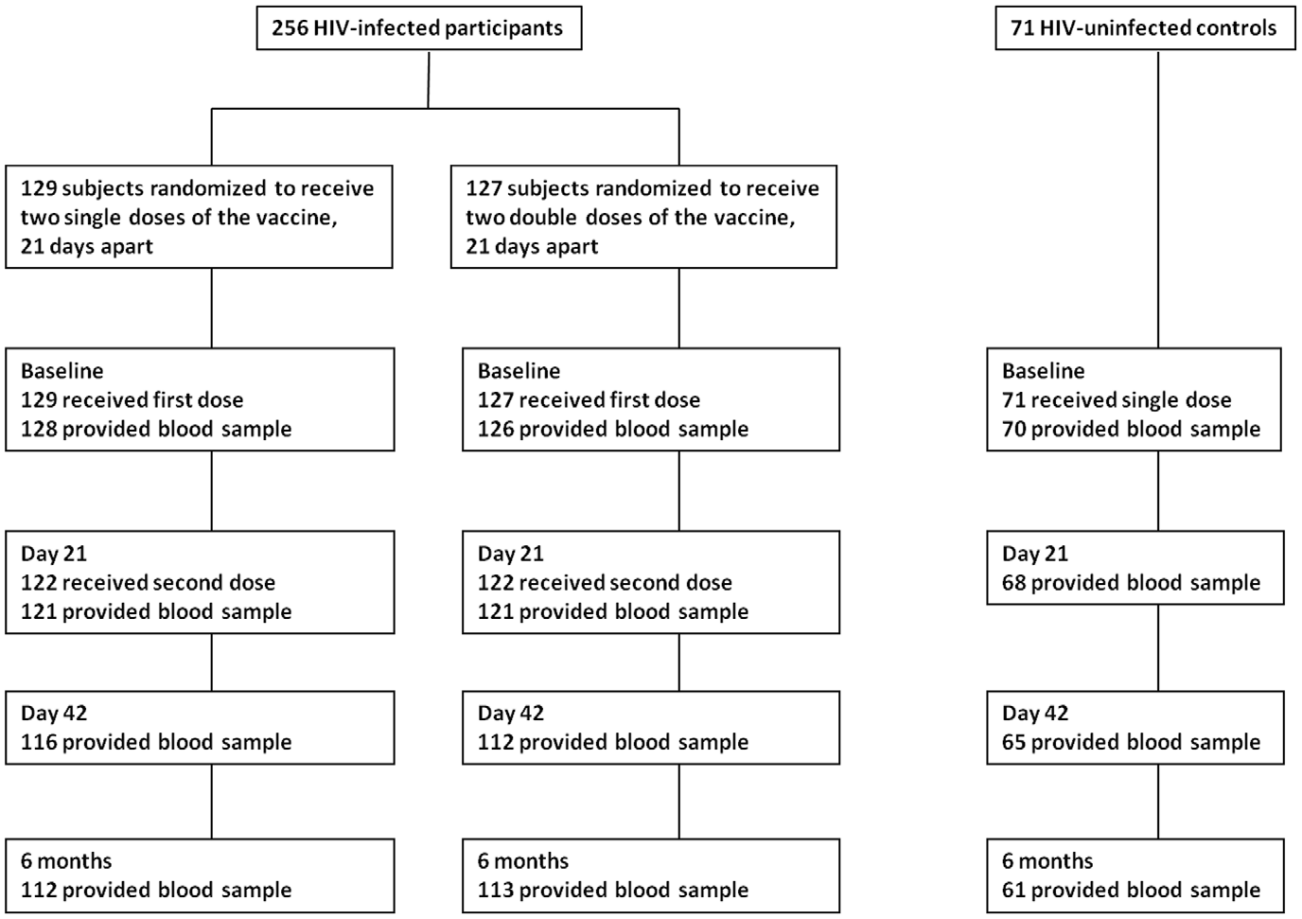
Participant disposition: enrollment, study procedures and participant disposition.

Two-hundred and fifty six patients were randomized, 129 received two single doses and 127 received two double doses (Figure 1). All 256 patients received the first dose of the vaccine, and 95.3% (244) received the second dose. Withdrawals were due to clinical conditions detected after recruitment (1 patient) and missed appointments (11 patients). Among the 71 controls, there were 3 missed appointments for health-unrelated reasons.

**Figure 2:**
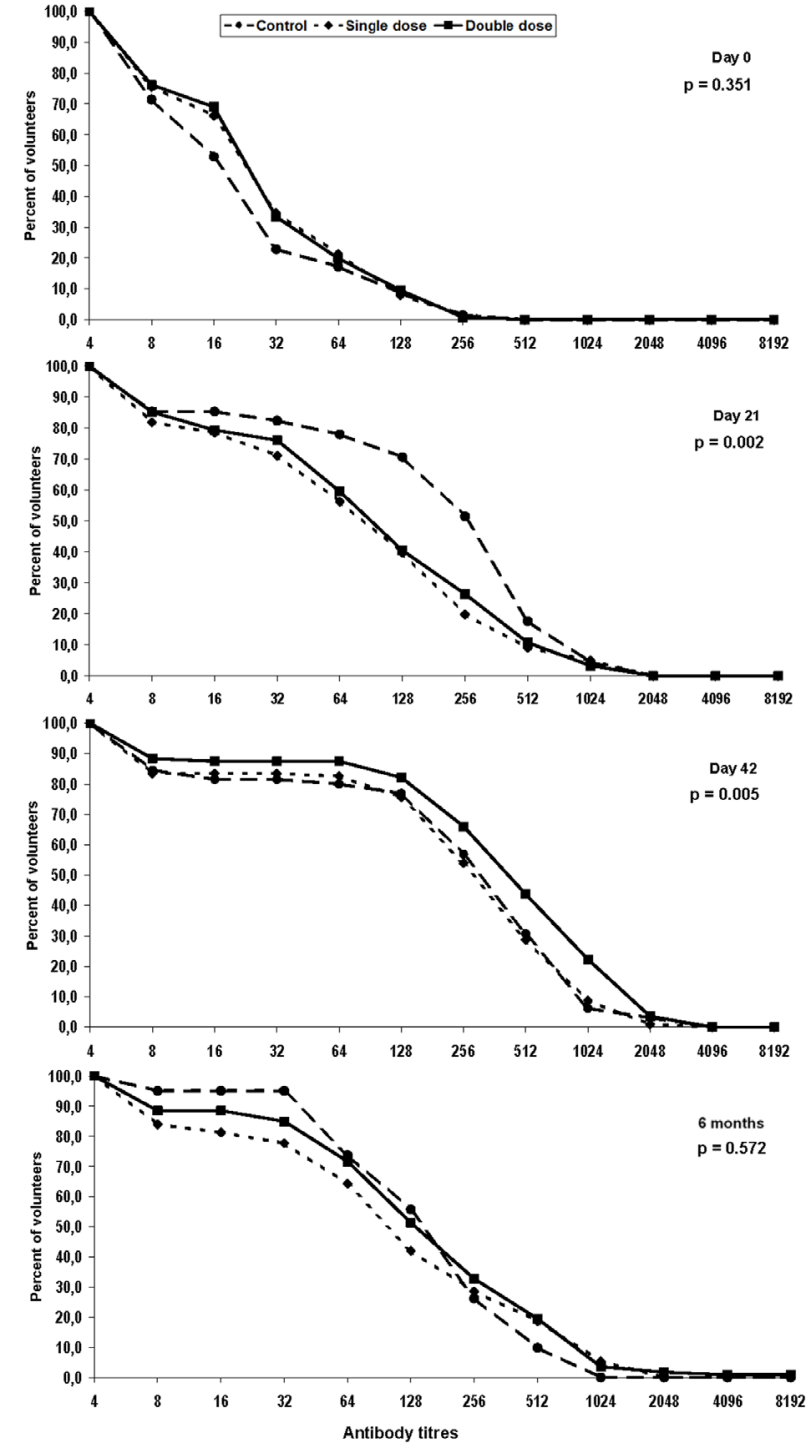
Reverse cumulative distribution curves of HAI antibody titers in samples collected on days 0, 21, 42, and at 6 months by vaccine group of participants: HIV-infected who received single doses (dashed line), HIV-infected who received double doses (solid line), and HIV-negative (dotted line). Values are given on a log scale.

Patients showed a balanced distribution of baseline demographic and clinical variables (Table 1) including pre-vaccination antibody levels (Table 2 and Figure 2, “Day 0” plot). Patients were predominantly middle-aged adult men, whereas among controls younger females prevailed. At enrollment, almost 80% of the patients were on HAART, CD4 cell counts surpassed 300 cells/mm^3^ for more than 75% of them and approximately 65% had undetectable HIV RNA viral load (Table 1). Approximately half of the patients had a nadir CD4 cell count below 200 cells/mm^3^.

**Table 1:** Baseline demographic and clinical characteristics of the study population.

Characteristic	HIV-positiveSingle dose	HIV-positiveDouble dose	HIV-negative
Number of participants	129	127	71
Age			
Median (IQR)	41.7 (36.0, 47.6)	42.8 (34.9, 50.1)	33.7 (29.1, 47.0)
Mean (SD)	42.2 (8.5)	42.0 (9.2)	37.3 (10.5)
≤40 years, N (%)	60 (46.5)	52 (40.9)	46 (64.8)
>40 years, N (%)	69 (53.5)	75 (59.1)	25 (35.2)
Gender			
Male, N (%)	90 (69.8)	79 (62.2)	25 (35.2)
Current smoker			
Yes, N (%)	37 (28.7)	27 (21.3)	9 (12.7)
BMI			
≥25 Kg/m^2^, N (%)	58 (45.0)	62 (48.8)	42 (59.2)
Years since HIV diagnosis			NA
Median (IQR)	6.9 (3.1, 12.4)	5.9 (2.4, 12.9)	
Mean (SD)	8.0 (5.7)	8.0 (6.6)	
Previous AIDS-defining illness			NA
Yes, N (%)	56 (43.4)	51 (40.1)	
CD4 count (cells/mm^3^)			NA
Median (IQR)	567 (329, 758)	550 (356, 743)	
Mean (SD)	583.5 (327.3)	584.5 (310.8)	
Nadir CD4 count (cells/mm^3^)			NA
Median (IQR)	165 (70, 291)	205 (48, 325)	
Mean (SD)	222.3 (221.2)	226.8 (202.6)	
HIV RNA viral load			NA
<50 copies/ml, N (%)	84 (68.3)	78 (63.4)	
Missing, N (%)	6 (4.7)	4 (3.1)	
HAART			NA
Currently taking, N (%)	103 (79.8)	101 (79.5)	
Never received HAART, N (%)	20 (15.5)	21 (16.5)	
Not on HAART, N (%)	6 (4.7)	4 (3.1)	
Missing, N (%)	0 (0.0)	1 (0.8)	
Years on HAART			NA
Median (IQR)	4.6 (2.2, 9.9)	3.9 (1.8, 10.3)	
Mean (SD)	6.1 (4.8)	6.4 (5.5)	

IQR: interquartile range, BMI: body-mass index, HIV: human immunodeficiency virus, HAART: highly active antiretroviral therapy, NA: not applicable.

**Table 2:** Antibody responses to the different vaccine schedules among HIV-infected and HIV-uninfected participants according to time since vaccination.

	HIV-infected				HIV-uninfected		
	Single dose		Double dose		Single dose		
	N	Estimates	N	Estimates	N	Estimates	
**Baseline**							
Seroprotection*, N (%)	127	44 (34.6)	126	42 (33.3)	70		16 (22.9)
Age < = 40 years	58	18 (31.0)	51	13 (25.5)	45		11 (24.4)
Age >40 years	69	26 (37.7)	75	29 (38.7)	25		5 (20.0)
GMT (95% CI)	127	16.8 (13.9, 20.4)	126	17 (14.0, 20.6)	70		13.4 (10.3, 17.4)
Age < = 40 years	58	15.4 (11.8, 20.2)	51	12.9 (9.5, 17.4)	45		13.5 (9.5, 19.1)
Age >40 years	69	18.0 (13.7, 23.8)	75	20.5 (16.2, 26.1)	25		13.2 (9.0, 19.3)
**Day 21 (21 days after 1st dose)**							
Seroprotection (N. %)	121	86 (71.1)	121	92 (76.0)	68		56 (82.4)
Age < = 40 years	55	34 (61.8)	48	30 (62.5)	43		34 (79.1)
Age >40 years	66	52 (78.8)	73	62 (84.9)	25		22 (88.0)
Seroconversion** (N. %)	120	56 (46.7)	120	62 (51.7)	67		50 (74.6)
Age < = 40 years	54	23 (42.6)	47	22 (46.8)	42		29 (69.0)
Age >40 years	66	33 (50.0)	73	40 (54.8)	25		21 (84.0)
GMT (95% CI)	121	48.9 (36.8, 65.0)	121	56.1 (42.4, 74.3)	68		107.6 (73.2, 158.3)
Age < = 40 years	55	38.2 (24.6, 59.3)	48	33.9 (21.4, 53.8)	43		103.8 (61.9, 174.0)
Age >40 years	66	60.1 (41.6, 86.9)	73	78.1 (55.9, 109.2)	25		114.6 (64.8, 202.4)
**Day 42 (21 days after 2nd dose)**							
Seroprotection (N. %)	115	96 (83.5)	112	98 (87.5)	65		53 (81.5)
Age < = 40 years	51	41 (80.4)	41	32 (78.0)	40		31 (77.5)
Age >40 years	64	55 (85.9)	71	66 (93.0)	25		22 (88.0)
Seroconversion (N. %)	114	88 (77.2)	111	93 (83.8)	64		46 (71.9)
Age < = 40 years	50	38 (76.0)	40	30 (75.0)	39		25 (64.1)
Age >40 years	64	50 (78.1)	71	63 (88.7)	25		21 (84.0)
GMT (95% CI)	115	128.8 (94.0, 176.5)	112	206.1 (150.5, 282.5)	65		129.4 (84.1, 199.1)
Age < = 40 years	51	104.4 (64.1, 170.0)	41	121.7 (66.0, 224.4)	40		105.8 (60.2, 185.8)
Age >40 years	64	152.2 (101.0, 229.5)	71	279.5 (200.4, 389.8)	25		178.5 (92.1, 345.9)
**6 months after 1^st^ dose**							
Seroprotection (N. %)	112	87 (77.7)	113	96 (85.0)	61		58 (95.1)
Age < = 40 years	48	38 (79.2)	42	32 (76.2)	37		34 (91.9)
Age >40 years	64	49 (76.6)	71	64 (90.1)	24		24 (100.0)
GMT (95% CI)	112	64.8 (47.6, 88.1)	113	86.9 (65.2, 115.9)	61		91.0 (68.7, 120.6)
Age < = 40 years	48	66.8 (41.2, 108.3)	42	66.1 (38.4, 113.9)	37		94.8 (62.7, 143.3)
Age >40 years	64	63.3 (42.4, 94.5)	71	102.2 (74.1, 141.1)	24		85.4 (61.0, 119.6)
Persistence of seroconversion*** (N. %)	85	68 (80.0)	89	80 (89.9)	45		45 (100)
Age < = 40 years	36	30 (83.3)	28	24 (85.7)	25		25 (100)
Age >40 years	49	38 (77.6)	61	56 (91.8)	20		20 (100)

HIV: human immunodeficiency virus, GMT: geometric mean titer.

* Hemagglutination inhibition assay (HAI) titre >1∶32.

** HAI titer ≤1∶8 before and at least 1∶32 after vaccination or baseline HAI titer >1∶8 and at least 4 fold increase after vaccination.

*** Persistence of seroprotective titers among patients who seroconverted on day 42.

At baseline, more than 1/3 of the patients and 1/5 of the controls had seroprotective antibody levels (i.e. ≥1∶32, Table 2). Antibody levels and the proportion seroprotected were slightly higher in the subgroup aged >40 years. Twenty one days after the first dose, the proportion seroprotected, antibody levels and geometric mean titer (GMT) rose substantially in all groups (Table 2 and Figure 2, Day 21). Among patients, the rise implied approximately 50% seroconversion (slightly higher among recipients of double doses) (Table 2). Seroconversion and antibody levels were much higher among controls, despite the modest seroprotection (Table 2 and Figure 2, Day 21). In all groups seroconversion rates and GMT following the first dose were somewhat higher among participants aged >40 years.

After the second dose, patients experienced a substantial rise of seroprotection and seroconversion, which exceeded that of controls. Antibody levels among those receiving double doses far exceeded that of controls (Table 2 and Figure 2, Day 42). Participants aged >40 years showed higher seroconversion rates and higher GMT, compared to younger participants, particularly among controls and patients who received double doses.

Six months (140–196 days) after recruitment, seroprotection rates among patients decreased slightly and GMT fell to half the level on day 42. Among controls, seroprotection rates increased substantially but the GMT decreased about 30% compared to day 42 levels (Table 2).

Seroconversion rates and GMT were consistently lower in the single dose group, at all times, and the confidence limits of the difference in proportions of seroconversion and GMT ratios did not provided evidence to reject the hypothesis of inferiority of the single dose (Table 3). In fact, superiority of two *double* doses is suggested by a GMT exceeding the upper 95% confidence limit of the GMT after two *single* doses (Table 2) and the statistical significance (Log-rank, p = 0.003) of cumulative curves for double and single doses (Figure 2, Day 42).

**Table 3:** Differences in proportions of seroconversion and seroprotection and ratio of GMT for single dose when compared to double dose according to time since vaccination.

	Differences/ratios and confidence intervals
**Day 21 (21 days after first dose)**	
Seroprotection*	(P_single_ – P_double_) and 95% CI
Overall	−5.0 (−16.9, 7.0)
Age < = 40 years	−0.7 (−21.4, 20.0)
Age >40 years	−6.1 (−20.4, 8.1)
Seroconversion**	(P_single_ – P_double_) and 95% CI
Overall	−5.0 (−18.5, 8.5)
Age < = 40 years	−4.2 (−25.6, 17.2)
Age >40 years	−4.8 (−22.8, 13.3)
GMT	GMT_single_/GMT_double_ and 95% CI
Overall	0.87 (0.58, 1.30)
Age < = 40 years	1.13 (0.59, 2.15)
Age >40 years	0.77 (0.47, 1.27)
**Day 42 (21 days after second dose)**	
Seroprotection	(P_single_ – P_double_) and 95% CI
Overall	−4.0 (−14.0, 6.0)
Age < = 40 years	2.3 (−16.6, 21.3)
Age >40 years	−7.0 (−18.9, 4.9)
Seroconversion	(P_single_ – P_double_) and 95% CI
Overall	−6.6 (−17.8, 4.6)
Age < = 40 years	1.0 (−19.1, 21.1)
Age >40 years	−10.6 (−24.6, 3.4)
GMT	GMT_single_/GMT_double_ and 95% CI
Overall	0.62 (0.40, 0.98)
Age < = 40 years	0.86 (0.39, 1.88)
Age >40 years	0.54 (0.32, 0.92)
**Six months after 1^st^ dose**	
Seroprotection	(P_single_ – P_double_) and 95% CI
Overall	−7.3 (−18.3, 3.8)
Age < = 40 years	3.0 (−16.5, 22.5)
Age >40 years	−13.6 (−27.5, 0.4)
GMT	GMT_single_/GMT_double_ and 95% CI
Overall	0.75 (0.49, 1.14)
Age < = 40 years	1.01 (0.48, 2.11)
Age >40 years	0.62 (0.37, 1.04)
Persistence of seroconversion***	(P_single_ – P_double_) and 95% CI
Overall	−9.9 (−21.6, 1.8)
Age < = 40 years	−2.4 (−23.3, 18.6)
Age >40 years	−14.3 (−29.7, 1.1)

* HAI titre >1∶32.

** HAI titer ≤1∶8 before and at least 1∶32 after vaccination or baseline HAI titer >1∶8 and at least 4 fold increase after vaccination.

*** Persistence of seroprotective titers among patients who seroconverted on day 42.

Compared to HIV-negative controls, seroconversion after the second dose occurred 1.82 times more often among two double doses recipients but the association lacked statistical significance (Table 4). Older age (>40 years) also increased the odds of seroconversion, being the only statistically significant predictor after adjustment of covariates. Consistently, the differences in antibody titers between HIV-infected groups and HIV-negative controls 42 days after vaccination adjusted for antibody titer before vaccination and for age were not statistically significant (data not shown).

**Table 4:** Demographic and clinical factors associated with the unadjusted and adjusted odds of seroconversion (odds ratio, OR, and 95% confidence interval, 95% CI).

	N	Unadjusted OR (95%CI)	Adjusted OR (95%CI)
**Seroconversion**			
Age <40 years (vs. ≥40 years)	290	0.49 (0.28, 0.86)	0.54 (0.30, 0.98)
Male (vs. Female)	290	0.96 (0.55, 1.71)	0.90 (0.49, 1.64)
BMI (per unit increase in BMI)	290	1.03 (0.97, 1.10)	1.05 (0.98, 1.12)
Current smoker (vs. not)	290	1.74 (0.83, 3.64)	1.80 (0.83, 3.92)
HIV+, Single dose (vs. HIV−)	290	1.28 (0.64, 2.56)	1.15 (0.55, 2.41)
HIV+, Double dose (vs. HIV−)	290	2.02 (0.96, 4.25)	1.82 (0.83, 3.98)

BMI: body-mass index, HIV: human immunodeficiency virus.

Among patients, seroconversion provided by two double doses was higher for those aged >40, women, those with nadir CD4 cell count <200 cells/mm^3^, with undetectable viral load, and on HAART (Table 5). Seroconversion among women and those with nadir CD4 cell count <200 cells/mm^3^ was significantly higher when two double doses were used.

**Table 5:** Proportion of HIV-infected individuals who seroconverted (as measured on day 42 after the first vaccine dose) as a function of demographic and clinical factors and vaccine schedule.

	Singledose % (N)	Doubledose % (N)	p-value*
**Seroconversion**			
Age			
≤40 years	74.5 (38/51)	75 (30/40)	1.000
>40 years	78.1 (50/64)	88.7 (63/71)	0.108
Sex			
Male	80.0 (64/80)	81.2 (56/69)	1.000
Female	68.6 (24/35)	88.1 (37/42)	0.049
Current smoker			
No	74.4 (61/82)	81.6 (71/87)	0.271
Yes	81.8 (27/33)	91.7 (22/24)	0.255
BMI			
<25	75.8 (47/62)	85.5 (47/55)	0.246
≥25	77.4 (41/53)	82.1 (46/56)	0.351
Baseline CD4 cell count (cells/mm^3^)			
<350	78.1 (25/32)	87.5 (21/24)	0.489
≥350	75.9 (63/83)	82.8 (72/87)	0.343
Nadir CD4 cell count (cells/mm^3^)			
<200	70.8 (46/65)	88.7 (47/53)	0.023
≥200	84.0 (42/50)	79.3 (46/58)	0.623
Baseline viral load (copies/ml)			
<50	77.9 (60/77)	88.4 (61/69)	0.124
≥50	73.5 (25/34)	76.3 (29/38)	0.793
Currently taking HAART			
No	72.7 (16/22)	66.7 (14/21)	0.747
Yes	77.4 (72/93)	87.8 (79/90)	0.080

* p-values based on Fisher’s exact test.

The average reduction in CD4 cell counts 21 days after the first dose was more pronounced among participants who received two double doses, but the difference was not statistically significant nor clinically relevant (Table 6). After the second dose, a small average increase in CD4 cell counts was observed for the single dose group while the double dose group showed a negligible difference. In groups, small and similar percentages of patients showed increased and decreased HIV viral load.

**Table 6:** Effect of vaccination on plasma HIV RNA level and CD4 cell count as given by the change in these parameters, from baseline, with respect to different times after vaccination.

	HIV-infected	HIV-infected	
	Single dose	Double dose	p-value
**21 days after first dose**			
Δ CD4^a^ cells/mm^3^, mean (SE)	−4.3 (15.2)	−33.8 (18.8)	0.226
Δ Log viral load^b^ copies/ml, mean (SE)	0.14 (0.04)	0.17 (0.04)	0.598
Viral load unchanged, N (%)	105 (89.7)	104 (87.4)	0.852
Viral load increased^c^, N (%)	8 (6.8)	10 (8.4)	
Viral load decreased, N (%)	4 (3.4)	5 (4.2)	
**42 days after first dose**			
Δ CD4^a^ cells/mm^3^, mean (SE)	17.8 (22.1)	1.1 (21.6)	0.590
Δ Log viral load^b^ copies/ml, mean (SE)	0.32 (0.06)	0.34 (0.06)	0.893
Viral load unchanged, N (%)	88 (79.3)	82 (76.6)	0.615
Viral load increased^c^, N (%)	10 (9.0)	14 (13.1)	
Viral load decreased, N (%)	13 (11.7)	11 (10.3)	

Log: log base 10, SE: standard error.

a Difference between CD4 cell counts at each time and baseline CD4 cell count.

b Difference between Log viral load at each time and baseline Log viral load.

c Increase is defined as a change from undetectable to detectable or, if detectable, a 0.5 log increase.

**Figure 3:**
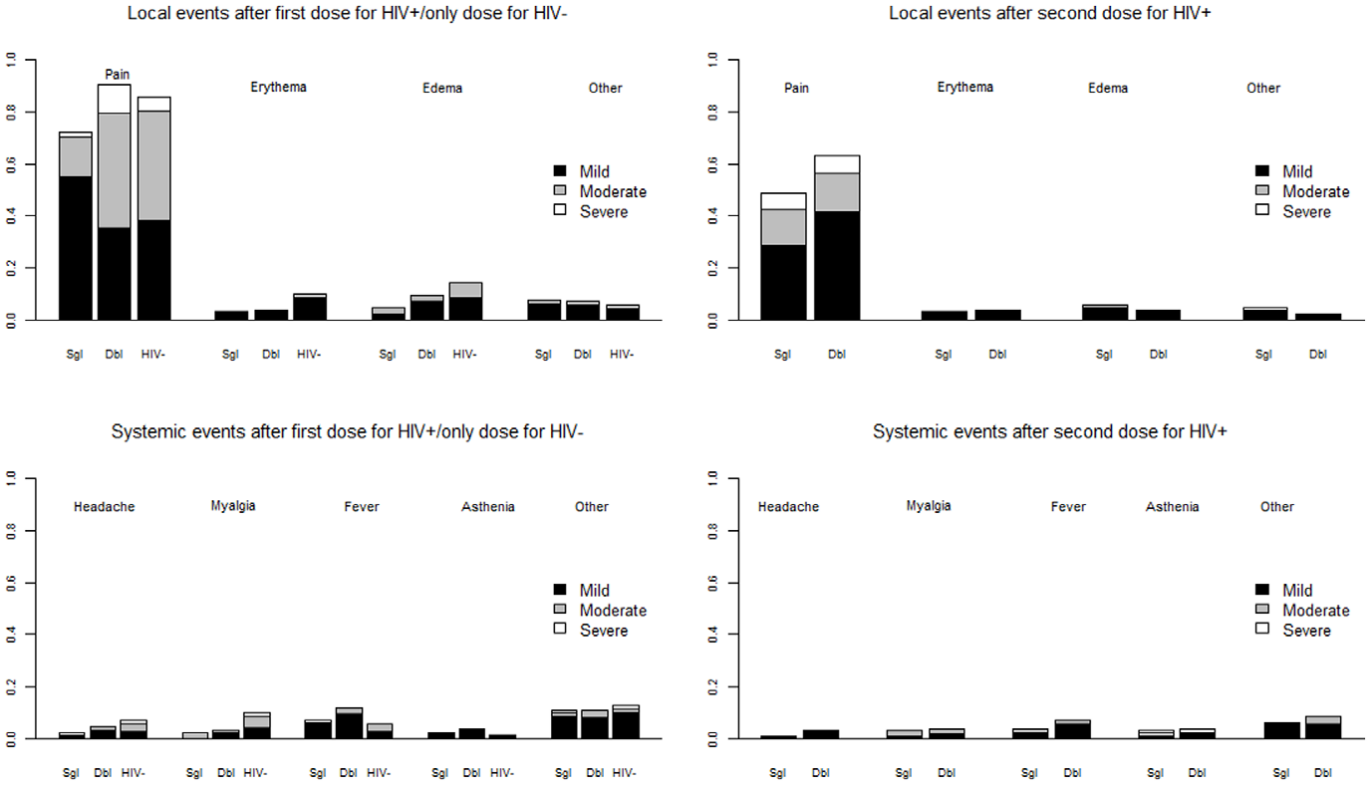
Adverse events: local and systemic adverse events 21 days after first dose and 42 days after the first dose in the three groups: HIV-infected who received single doses, HIV-infected who received double doses, and HIV-negative.

The most frequent local adverse reaction was pain, reported after the first dose, in 72% and 91% of the patients who received single and double doses, respectively (Figure 3). In these same groups, after the second dose, 52% and 66% of the participants reported pain. Eighty-six percent of the controls also reported pain. The most frequent systemic adverse event was fever, which was reported, after the first dose, by 7% and 12% of the patients who received single and double doses, respectively.

A total of 43 participants presented with ILI within 6 months of the first vaccine dose: 14 and 17 ILI events in patients receiving single and double doses, respectively, and 10 ILI events among controls. Nasopharyngeal samples were collected for 22 participants (Table 7). No H1N1pdm09-related ILI was identified. In six patients seasonal influenza A H3N2 (2 patients) or influenza B (4 patients) viruses were detected, which is in line with the influenza season that occurred during the period of study. Of note, one patient that received double doses was co-infected with both rhinovirus and influenza B.

**Table 7:** Etiological agent causing the ILI event.

Group	Etiological agent	Number of samples
HIV-infected single dose	Influenza B	2
HIV-infected single dose	Coronavirus 63	1
HIV-infected single dose	Influenza A (H3N2)	1
HIV-infected single dose	Rhinovirus	1
HIV-infected single dose	Metapneumovirus	1
HIV-infected single dose	Coravirus HK	1
HIV-infected double dose	Influenza A (H3N2)	1
HIV-infected double dose	Rhinovirus	1
HIV-infected double dose	Coronavirus 63	1
HIV-infected double dose	Influenza B	1
HIV-infected double dose	Rhinovirus and Influenza B	1
HIV-infected double dose	Metapneumovirus	1
Control	Coronavirus 43	1
Control	Rhinovirus	1
**Total**		**15**

No etiological agent was identified in 7 samples (3 HIV-infected double doses, 2 HIV-infected single doses, and 3 controls).

## Discussion

To our knowledge, this is the first randomized, controlled trial designed to evaluate efficacy and sustainability of protective titers of an adjuvanted H1N1pdm09 vaccine in HIV-infected adults in Latin America. From April through December 2009, during the fall/winter of the southern hemisphere, the H1N1pdm09 virus circulated in Brazil [16], and, by the end of 2009, over 2000 H1N1pdm09-related deaths were reported [9]. International and local guidelines recommended prioritization of vaccination against H1N1pdm09 for HIV-infected individuals [4], [5]. Vaccination was carried out with a single dose, following the schedule for HIV-negative individuals. However, seroconversion to influenza vaccines among HIV-infected individuals have been shown to be lower than those observed in the general population [17], [18]. In light of this scenario, we evaluated an alternative vaccination scheme for HIV-infected individuals.

In our HIV-infected population, seroconversion increased from 47% and 52% after the first dose to 77% and 84% after the second dose of the vaccine using single and double doses, respectively. Similarly, seroprotection increased from 71% and 76% after the first dose to 84% and 88% after the second dose of the same vaccination schemes. These percentages of seroconversion are comparable to those found by Soonawala et al [19] in a smaller and non-randomized study and lower than the seroconversions found by Bickel [6] and Launay [7] who enrolled asymptomatic, HIV-infected adults in Germany and France. In these studies, most patients’ characteristics were comparable to those from our study population (ie, high mean baseline CD4 cell count). In contrast, although HAART was used by almost 80% of our patients, undetectable viral load was observed in roughly 65% and 40% had a previous AIDS-defining illness. That is, in comparison to the other studies cited above, our study population had more advanced disease.

In addition, a different pattern of H1N1pdm09 virus circulation in the northern and southern hemispheres in 2009/2010 may hamper the comparison of our seroconversion results with those of others [6], [7], [19]. Studies from the northern hemisphere enrolled patients from November to December of 2009, which overlapped with the second wave of the H1N1pdm09 pandemic in these locations [20]. Since circulating and vaccine strains of H1N1pdm09 virus were virtually the same, sub-clinical infection with the circulating viruses may have boosted the immune response in individuals from the northern hemisphere, leading to higher HA titers. In Brazil, in contrast, vaccination was carried out prior to the southern hemisphere’s winter of 2010. H1N1pdm09 circulation in 2010 in Brazil decreased significantly when compared to 2009 [20]. In fact, the Brazilian surveillance system detected influenza A H3N2 and B circulation in 2010, but no concomitant H1N1pdm09 circulation [20]. Thus, there was minimal H1N1pdm09 circulation during our study and, consequently, immune boosts due to virus circulation were unlikely. In our study, one third of the HIV-infected participants had seroprotective antibody titers at baseline. This value is higher than that observed in other H1N1pdm09 studies conducted in the northern hemisphere [6], [7], corroborating the high H1N1pdm09 circulation in 2009 in Brazil [9].

Our results showed that the level of protective titers (among seroconverters) six months after vaccination was lower in patients when compared to controls. These results are consistent with other reports of decreased sustained antibody response among HIV-infected adults [21]. Data on the durability of influenza antibodies after seasonal vaccination for the general population suggest that protection lasts for at least one year [22], [23]. For the HIV-infected population, very limited data is available as most studies only report 30-days of follow-up. Sustainability of antibody response is important since the influenza season often spans an estimated six months [24]. Furthermore, pandemic influenza infections may occur during non-seasonal months necessitating prolonged immunity [25]. Our study shows that a higher proportion of the patients who received two double doses were able to sustain protective antibody responses at six months. Thus, our data suggest that a modified vaccination schedule may confer higher sustained seroprotection. This finding has important implications for both HIV care and public health policy.

Our results suggest that two double doses of an adjuvanted H1N1pdm09 vaccine elicited a significantly higher seroconversion among women when compared to two single doses. Historically, women were underrepresented in HIV clinical trials of antiretroviral therapy, as well as in other therapeutic trials in the areas of cardiovascular, lung, and cancer research [26], [27], [28]. Currently, there is increased awareness of the need for representative inclusion of women in HIV clinical trials. More than one third of our participants were women, a higher percentage when compared to all trials previously published on H1N1pdm09 vaccination among HIV-infected individuals. It is likely that our study is the first to evaluate a potential sex-based difference in seroconversion as a function of vaccination schemes, being thus a unique finding of our study.

The increased seroconversion among participants aged >40 years might be due to previous exposure to a low glycosylated H1N1 virus during their lifetime. A series of publications have described the similarities between the hemagglutinin from the 1918 and 2009 viruses, which are both low glycosylated [29], [30], [31]. Although more glycosylated than those from 1918 and 2009, viruses from the 1970s are also more similar to these agents than to viruses that circulated in 1990/2000 that are heavily glycosylated. Therefore, previous exposure to viruses from the 1970s could confer cross-immunity to H1N1pdm09. Consequently, it is possible that vaccination against H1N1pdm09 could have boosted an existing immunity to low glycosylated hemagglutinins, which is more likely to be present in participants aged >40 years.

During the six months of follow-up, there were 43 ILI out of which 22 were laboratory diagnosed. Fifteen participants had at least one respiratory virus detected, but no H1N1pdm09 cases were found. The vaccine was well tolerated in our study population. Local pain and fever, which were the most frequent local and systemic adverse events, were more frequently seen among patients randomized to the double dose arm. In regard to the impact of vaccination on HIV viral load and CD4 cell counts, our results corroborate recent findings of no clinically meaningful effect of vaccination on CD4 cell counts and HIV viral load [6], [21], [32]. We also did not find an association of seroconversion with baseline CD4 cell counts. Our results show that double doses elicited significant higher seroconversion among patients with nadir CD4 cell counts <200 cells/mm^3^.

Limitations of our study should be acknowledged. This was a single-center study, with entry criteria limited to those patients without current comorbidities, and conducted over a single H1N1pdm09 season. We evaluated a well controlled cohort of HIV-infected individuals and, thus, could not determine the impact of current severe immunosuppression on H1N1pdm09 vaccine responses. However, our study had the advantage of concurrently evaluating a group of HIV-uninfected controls and a significant proportion of women. Our study provides compelling evidence for the need of a different vaccination scheme for HIV-infected individuals. A higher vaccine dose would provide additional benefits for women and for those with a history of advanced immunodeficiency.

In summary, our results show that patients only achieved levels of seroconversion comparable to those of controls after two doses of an adjuvanted vaccine. The 21-days interval between doses was more important in inducing higher seroconversion than the vaccine hemagglutinin dosage. Patients with nadir CD4 cell counts <200 and women showed improved seroconversion with double doses, which also provided more sustained seroprotection. Our findings contribute to the planning of next year’s influenza vaccination campaign also by suggesting that if the vaccine is used at the currently recommended dosage and schedule, a significant proportion of the individuals will remain vulnerable to influenza.

## Supporting Information

Checklist S1 **CONSORT Checklist.**
(DOC)

Protocol S1 **Trial Protocol.**
(PDF)

## References

[1] Beck JM, Rosen MJ, Peavy HH (2001). Pulmonary complications of HIV infection. Report of the Fourth NHLBI Workshop.. Am J Respir Crit Care Med.

[2] Bautista E, Chotpitayasunondh T, Gao Z, Harper SA, Shaw M (2010). Clinical aspects of pandemic 2009 influenza A (H1N1) virus infection.. N Engl J Med.

[3] Klein MB, Lu Y, DelBalso L, Cote S, Boivin G (2007). Influenzavirus infection is a primary cause of febrile respiratory illness in HIV-infected adults, despite vaccination.. Clin Infect Dis.

[4] CDC (2011). Updated Interim Recommendations - HIV-Infected Adults and Adolescents: Considerations for Clinicians Regarding 2009 H1N1 Influenza.. http://www.cdc.gov/h1n1flu/guidance_hiv.htm.

[5] MS (2010). Nota técnica 67/2010: Pessoas que vivem com HIV/aids serão vacinadas contra o Vírus da Influenza A (H1N1). Departamento de DST, AIDS e Hepatites Virais, Ministério da Saúde.. http://www.aids.gov.br/node/40142.

[6] Bickel M, von Hentig N, Wieters I, Khaykin P, Nisius G (2011). Immune response after two doses of the novel split virion, adjuvanted pandemic H1N1 influenza A vaccine in HIV-1-infected patients.. Clin Infect Dis.

[7] Launay O, Desaint C, Durier C, Loulergue P, Duval X (2011). Safety and immunogenicity of a monovalent 2009 influenza A/H1N1v vaccine adjuvanted with AS03A or unadjuvanted in HIV-infected adults: a randomized, controlled trial.. J Infect Dis.

[8] Dolin R (2011). Editorial commentary: Perspectives on the role of immunization against influenza in HIV-infected patients.. Clin Infect Dis.

[9] Oliveira W, Carmo E, Penna G, Kuchenbecker R, Santos H (2009). Pandemic H1N1 influenza in Brazil: analysis of the first 34,506 notified cases of influenza-like illness with severe acute respiratory infection (SARI).. Euro Surveill 14.

[10] Miller E, Hoschler K, Hardelid P, Stanford E, Andrews N (2010). Incidence of 2009 pandemic influenza A H1N1 infection in England: a cross-sectional serological study.. Lancet.

[11] Clark TW, Pareek M, Hoschler K, Dillon H, Nicholson KG (2009). Trial of 2009 influenza A (H1N1) monovalent MF59-adjuvanted vaccine.. N Engl J Med.

[12] Rowe T, Abernathy RA, Hu-Primmer J, Thompson WW, Lu X (1999). Detection of antibody to avian influenza A (H5N1) virus in human serum by using a combination of serologic assays.. J Clin Microbiol.

[13] Szretter KJ, Balish AL, Katz JM (2006). Influenza: propagation, quantification, and storage.. Curr Protoc Microbiol Chapter 15: Unit 15G 11.

[14] WHO (2009). CDC protocol for realtime RTPCR for influenza A (H1N1), World Health Organization.. http://www.who.int/csr/resources/publications/swineflu/CDCRealtimeRTPCR_SwineH1Assay-2009_20090430.pdf.

[15] Fleiss JL (1981). Statistical methods for rates and proportions. New York: Wiley.. xviii, 321 p.

[16] SVS (2009). Informe epidemiológico influenza A H1N1: Situação epidemiológica da nova influenza A (H1N1) no Brasil. Secretaria de Vigilancia em Saude.. http://portal.saude.gov.br/portal/arquivos/pdf/informe_influenza_se30_03_08_2009.pdf.

[17] Bickel M, Wieters I, Khaykin P, Nisius G, Haberl A (2010). Low rate of seroconversion after vaccination with a split virion, adjuvanted pandemic H1N1 influenza vaccine in HIV-1-infected patients.. AIDS.

[18] Tebas P, Frank I, Lewis M, Quinn J, Zifchak L (2010). Poor immunogenicity of the H1N1 2009 vaccine in well controlled HIV-infected individuals.. AIDS.

[19] Soonawala D, Rimmelzwaan GF, Gelinck LB, Visser LG, Kroon FP (2011). Response to 2009 pandemic influenza A (H1N1) vaccine in HIV-infected patients and the influence of prior seasonal influenza vaccination.. PLoS One.

[20] WHO (2011). FluNet.. http://www.who.int/influenza/gisrs_laboratory/flunet/en/.

[21] Crum-Cianflone NF, Iverson E, Defang G, Blair PJ, Eberly LE (2011). Durability of antibody responses after receipt of the monovalent 2009 pandemic influenza A (H1N1) vaccine among HIV-infected and HIV-uninfected adults.. Vaccine.

[22] CDC (2011). Inactivated Influenza Vaccine: What you need to know, 2010–2011.. http://www.cdc.gov/vaccines/pubs/vis/#flu.

[23] Couch RB, Kasel JA (1983). Immunity to influenza in man.. Annu Rev Microbiol.

[24] Fiore AE, Uyeki TM, Broder K, Finelli L, Euler GL (2010). Prevention and control of influenza with vaccines: recommendations of the Advisory Committee on Immunization Practices (ACIP), 2010.. MMWR Recomm Rep.

[25] CDC (2011). Updated CDC Estimates of 2009 H1N1 Influenza Cases, Hospitalizations and Deaths in the United States, April 2009 – April 10, 2010.. http://www.cdc.gov/h1n1flu/estimates_2009_h1n1.htm.

[26] Harris DJ, Douglas PS (2000). Enrollment of women in cardiovascular clinical trials funded by the National Heart, Lung, and Blood Institute.. N Engl J Med.

[27] Jagsi R, Motomura AR, Amarnath S, Jankovic A, Sheets N (2009). Under-representation of women in high-impact published clinical cancer research.. Cancer.

[28] Melloni C, Berger JS, Wang TY, Gunes F, Stebbins A (2010). Representation of women in randomized clinical trials of cardiovascular disease prevention.. Circ Cardiovasc Qual Outcomes.

[29] Settembre EC, Dormitzer PR, Rappuoli R (2010). H1N1: can a pandemic cycle be broken?. Sci Transl Med.

[30] Wei CJ, Boyington JC, Dai K, Houser KV, Pearce MB (2010). Cross-neutralization of 1918 and 2009 influenza viruses: role of glycans in viral evolution and vaccine design.. Sci Transl Med.

[31] Xu R, Ekiert DC, Krause JC, Hai R, Crowe JE (2010). Structural basis of preexisting immunity to the 2009 H1N1 pandemic influenza virus.. Science.

[32] Crum-Cianflone NF, Eberly LE, Duplessis C, Maguire J, Ganesan A (2011). Immunogenicity of a monovalent 2009 influenza A (H1N1) vaccine in an immunocompromised population: a prospective study comparing HIV-infected adults with HIV-uninfected adults.. Clin Infect Dis.

