# Supplementary material for: H1N1pdm09 Adjuvanted Vaccination in HIV-Infected Adults: A Randomized Trial of Two Single versus Two Double Doses
Source: PLoS One. 2012 Jun 25;7(6):e39310. doi: 10.1371/journal.pone.0039310 (PMC3382468; doi:10.1371/journal.pone.0039310)
Supplement: Protocol S1 — Trial Protocol. (PDF) [file pone.0039310.s002.pdf]

PROTOCOLO CLÍNICO DE PESQUISA

Estudo de Fase II para avaliar a segurança e a imunogenicidade da vacina anti H1N1 em pacientes infectados pelo HIV

Versão 1.0 de 02 de Fevereiro de 2010

Investigador Principal **Beatriz Grinsztejn**

Co-Investigador Principal: **Luís Antonio Camacho**

INFORMAÇÃO CONFIDENCIAL

Este protocolo é propriedade dos pesquisadores aqui citados e contém informações confidenciais. O uso de todo o conteúdo é restrito, destinando-se a orientar os pesquisadores, suas equipes e colaboradores nos procedimentos a serem seguidos durante o estudo. Esse material não deve ser revelado ou usado por outros, exceto com autorização dos pesquisadores.

SUMÁRIO

Lista de abreviaturas	1
Centros que participam do estudo e equipe do protocolo	2
Gerenciamento do estudo	4
Esquema	6
1.0 Hipótese e objetivos do estudo	7
1.1 Hipóteses	7
1.2 Objetivos primários	7
1.3 Objetivos secundários	7
2.0 Introdução	8
2.1 Contexto	8
2.2 Justificativa	13
3.0 Desenho do estudo	16
4.0 Escolha e recrutamento dos participantes	17
4.1 Critérios de inclusão	17
4.2 Critérios de exclusão	18
4.3 Procedimentos de recrutamento no estudo	19
5.0 Tratamento do estudo	20
5.1 Esquemas e administração da vacina	20
5.1.1 Esquemas	20
5.1.2 Administração da vacina	20
5.2 Apresentação do produto do estudo e seu preparo	20
5.3 Farmácia: Fornecimento, distribuição e contabilidade do produto	21

5.4 Medicamentos concomitantes	22
6.0 Avaliações clínicas e laboratoriais	23
6.1 Cronograma de eventos	23
6.2 Momentos das avaliações	23
6.3 Instruções especiais e definições das avaliações	28
6.4 Estudo de custo-efetividade	33
7.0 Questões de gerenciamento clínico	34
7.1 Conduta nas reações no local de aplicação e nas reações alérgicas	34
7.2 Outros eventos adversos	34
7.2.1 Eventos Adversos Graves	35
7.3 Gestação	36
8.0 Critérios de retirada do estudo	37
8.1 Interrupção do esquema vacinal com permanência no estudo	37
8.2 Saída prematura do estudo	37
9.0 Considerações estatísticas	37
9.1 Questões gerais do desenho do estudo	37
9.2 Desfechos	38
9.3 Estratificação	38
9.4 Tamanho da amostra, seleção e recrutamento	38
9.5 Monitoramento	39
9.6 Análises	40
10.0 Plano farmacológico	41
10.1 Negociação com o Ministério da Saúde	41

10.2 Transporte do produto vacinal	41
10.3 Controle de estoque	41
10.4 Dispensação	41
11.0 Coleta de dados e controle e notificação de eventos adversos	41
11.1 Registros a serem mantidos	41
11.2 Papel do gerenciamento de dados	42
11.3 Monitoramento do centro clínico e disponibilidade dos registros	42
11.4 Eventos adversos de notificação imediata para o grupo coordenador médico do estudo	43
12.0 Participantes humanos	44
12.1 Revisão do Comitê de Ética em Pesquisa (CEP) e termo de consentimento livre e esclarecido	44
12.2 Confidencialidade da participante	44
12.3 Término do estudo	44
13.0 Publicação das descobertas da pesquisa	44
14.0 Contenção de risco biológico	44
15.0 Referências	45
Anexo I Fluxograma de procedimentos	
Anexo II Tabela para graduação de eventos adversos	
Anexo III Resumo das características do medicamento	
Anexo IV Termo de consentimento livre e esclarecido	
Anexo V Orçamento	
Anexo VI Cronograma de execução do estudo	

LISTA DE ABREVIATURAS

CEP	Comitê de Ética em Pesquisa
CTL	linfócitos T citotóxicos
CV	Carga viral do HIV
ENSP	Escola Nacional de Saúde Pública
EUA	Estados Unidos da América
GSK	Glaxo Smith & Kline
HÁ	Hemaglutinina
HAART	Terapia antirretroviral altamente potente (*highly active antiretroviral therapy*)
HAI	inibição da hemaglutinação (*hemagglutination inhibition)*
HAV	Vírus da hepatite A
HBV	Vírus da hepatite B
HIV	Vírus da Imunodeficiência Humana
IM	Intra-muscular
IOC	Instituto Oswaldo Cruz
IPEC	Instituto de Pesquisa Clínica Evandro Chagas
Nt	Neutralização
TARV	Terapia antirretroviral
TCLE	Termo de Consentimento Livre e Esclarecido
TIV	Vacina trivalente contra a influenza sazonal

CENTROS QUE PARTICIPAM DO ESTUDO E EQUIPE DO PROTOCOLO

Esse estudo será conduzido em um único centro de pesquisa, a Fundação Oswaldo Cruz (Fiocruz), no Rio de Janeiro-RJ. As seguintes unidades da Fiocruz estão envolvidas no projeto: Instituto de Pesquisa Clinica Evandro Chagas (IPEC), Instituto Oswaldo Cruz (IOC) e Escola nacional de Saúde Pública (ENSP)

Pesquisadores responsáveis:

Beatriz Grinsztejn
Laboratório de Pesquisa Clínica em DST e AIDS
Instituto de Pesquisa Clínica Evandro Chagas (IPEC) – Fiocruz
Av. Brasil 4365, Manguinhos, Rio de Janeiro, RJ. CEP 21040-360
Tel: (21) 2270-7064
gbeatriz@ipec.fiocruz.br

Luís Antonio Camacho
Departamento de Epidemiologia e Métodos Quantitativos em Saúde
Escola Nacional de Saúde Pública (ENSP) - Fiocruz
Av. Brasil 4365, Manguinhos, Rio de Janeiro, RJ. CEP 21040-360
Tel: (21) 2598 2630
luiz.camacho@ensp.fiocruz.br

Equipe do estudo

Marilia Santini de Oliveira (marilia.santini@ipec.fiocruz.br)
Valdilea Gonçalves Veloso (valdilea.veloso@ipec.fiocruz.br)
Maria Isabel Gouveia (isabel.gouveia@ipec.fiocruz.br)
Mauricio Teixeira Leite de Vasconcellos (mauricio.vasconcellos@ipec.fiocruz.br)
Thiago Torres (thiago.torres@ipec.fiocruz.br)
Ronaldo Ismério Moreira (ronaldo@fiocruz.br)
Paula Mendes Luz (paula.luz@ipec.fiocruz.br)
Estevão Portela Nunes (estevão.nunes@ipec.fiocruz.br)
Maria de Lourdes Benamor Teixeira (lourdes.benamor@ipec.fiocruz.br)
Alberto dos Santos de Lemos (alberto.lemos@ipec.fiocruz.br)
Laboratório de Pesquisa Clínica em DST e AIDS
Instituto de Pesquisa Clínica Evandro Chagas (IPEC) – Fiocruz
Av. Brasil 4365, Manguinhos, Rio de Janeiro, RJ. CEP 21040-360
Tel: (21) 2270-7064

Carmem Beatriz Wagner Giacoia Gripp
Mariza Gonçalves Morgado
Laboratório de AIDS e Imunologia Molecular
Instituto Oswaldo Cruz (IOC) - Fiocruz

Av. Brasil 4365, Manguinhos, Rio de Janeiro, RJ. CEP 21040-360
Tel: (21) 3865-8106

Marilda M. Siqueira (mmsiq@ioc.fiocruz.br)
Thiago Moreno L. Souza (tmoreno@ioc.fiocruz.br)
Fernando C. Motta (fcm@ioc.fiocruz.br)
Maria de Lourdes Oliveira (mlaoliveira@fiocruz.br)
Laboratório de Vírus Respiratório e do Sarampo
Instituto Oswaldo Cruz (IOC) - Fiocruz
Av. Brasil 4365, Manguinhos, Rio de Janeiro, RJ. CEP 21040-360

GERENCIAMENTO DO ESTUDO

Todas as perguntas referentes a este protocolo devem ser enviadas por e-mail para Beatriz Grinsztejn (gbeatriz@ipec.fiocruz.br) e Luiz Antonio Bastos Camacho (luiz.camacho@ensp.fiocruz.br). Em geral, a resposta deve ser recebida em 24 horas (de segunda a sexta-feira).

.Gerenciamento clínico

Para questões referentes a critérios de inclusão no estudo, conduta em casos de efeitos tóxicos, medicamentos concomitantes e co-recrutamento entrar em contato com Beatriz Grinsztejn (gbeatriz@ipec.fiocruz.br), Valdiléa Gonçalves Veloso dos Santos (valdilea.veloso@ipec.fiocruz.br), Marilia Santini de Oliveira (marilia.santini@ipec.fiocruz.br) e Maria Isabel Fragoso da Silveira Gouvêa (isabel.gouvea@ipec.fiocruz.br). Favor incluir no assunto da mensagem o título do protocolo, o número de identificação do paciente e um pequeno resumo relevante da história.

Laboratório

Para as questões relacionadas especificamente aos exames laboratoriais imunológicos, entrar em contato com os imunologistas do protocolo. Enviar uma mensagem de e-mail para Carmem Beatriz Wagner Giacoia Gripp (carmembg@ioc.fiocruz.br) e para Mariza Gonçalves Morgado (mmorgado@ioc.fiocruz.br).

Para as questões relacionadas especificamente à sorologia e aos exames laboratoriais de identificação viral, entrar em contato com o imunologista do protocolo. Enviar uma mensagem de e-mail para Marilda Siqueira (mmsiq@ioc.fiocruz.br).

Para as questões relacionadas especificamente aos exames laboratoriais de segurança, entrar em contato com Marilia Santini de Oliveira (marilia.santini@ipec.fiocruz.br) e Maria Isabel Fragoso da Silveira Gouvea (isabel.gouvea@ipec.fiocruz.br).

Gerenciamento de dados

Para questões não-clínicas acerca de transferências, critérios de inclusão/exclusão, fichas clínicas de estudo (*case report forms* – CRF), do cronograma de eventos do CRF, de randomização/registro, desvios e outras questões de gerenciamento de dados, entrar em contato com o Gerente de Dados, Ronaldo Ismério Moreira (ronaldo@fiocruz.br) .

Randomização

Para questões ou problemas de randomização e listas de número de identificação no estudo, entrar em contato com o gerente da central de dados, Ronaldo Ismério Moreira (ronaldo@fiocruz.br).

Tratamento do estudo

Para questões ou problemas relativos ao medicamento do estudo, posologia, suprimento, registros e devoluções entrar em contato com Thiago Torres, farmacêutico do Protocolo (thiago.torres@ipec.fiocruz.br).

Relato/questões sobre eventos adversos de notificação imediata (Eventos adversos graves – EAG)

Os eventos adversos graves devem ser reportados ao grupo coordenador médico do estudo, através dos e-mails Beatriz Grinsztejn (gbeatriz@ipec.fiocruz.br), Valdiléa Gonçalves Veloso dos Santos (valdilea.veloso@ipec.fiocruz.br), Marilia Santini de Oliveira (marilia.santini@ipec.fiocruz.br) e Maria Isabel Fragoso da Silveira Gouvea (isabel.gouvea@ipec.fiocruz.br). Favor incluir no assunto da mensagem o título do protocolo, o número de identificação do paciente e um pequeno resumo relevante da história.

ESQUEMA

Estudo de Fase II para avaliar a segurança e a imunogenicidade da vacina anti H1N1 em pacientes infectados pelo HIV

DESENHO

Este é um estudo randomizado, aberto, de fase II, para avaliar a segurança e a imunogenicidade de dois diferentes esquemas de vacinação contra a influenza A H1N1 em indivíduos infectados pelo HIV, no qual cada um dos grupos randomizados será comparado com voluntários HIV negativos vacinados com esquema indicado pelo Programa Nacional de Imunização.

DURAÇÃO

Cada participante permanecerá no estudo por cerca de 12 meses. A duração total do estudo será de 16 meses, considerando um período de inclusão de 4 meses.

POPULAÇÃO

Serão incluídos no estudo pacientes infectados pelo HIV, estratificados de acordo com a contagem de CD4 (≤ 200 cels/mm3 ou > 200 cels/mm3) no momento da triagem para o estudo, que estejam sem receber tratamento anti-retroviral ou em tratamento estável há no mínimo 8 semanas, sem planos de mudança nos próximos 6 meses, com indicação de receber vacina contra influenza A H1N1. O grupo controle será formado por indivíduos HIV negativos, status confirmado por sorologia realizada no momento da triagem, com indicação de receber vacina contra influenza A H1N1.

ESQUEMA

Os pacientes infectados pelo HIV receberão um de dois possíveis esquemas vacinais: 1) 3,75 µg em duas aplicações, com 21 dias de intervalo; 2) 7,5 µg em duas aplicações, com 21 dias de intervalo. Os voluntários do grupo controle receberão uma única aplicação da dose de 3,75 µg da vacina. Adicionalmente os participantes do estudo receberão vacina contra influenza sazonal no dia da segunda dose da vacina contra A H1N1 (portadores do HIV) ou 21 dias após a dose única da vacina contra A H1N1 (grupo controle).

TAMANHO DA AMOSTRA

Considerando um estudo de não-inferioridade com diferença aceitável de 10% e uma soroconversão de 95% foi determinado um tamanho de amostra de 82 pessoas por grupo, ampliado para 90 para lidar com perdas no processo, tendo em vista que os pacientes portadores do HIV serão selecionados aleatoriamente a partir do conjunto de pacientes em tratamento no IPEC. Em decorrência dos quatro grupos constituídos (estratos de contagem de CD4 e esquemas vacinais), serão incluídos no estudo 360 portadores de HIV e 90 HIV negativos, totalizando 450 voluntários no IPEC

1.0 HIPÓTESE E OBJETIVOS DO ESTUDO

1.1 Hipóteses

1. A vacina contra o H1N1 promove títulos de anticorpos acima do nível especificado para proteção (soroconversão), sendo tão segura e bem tolerada em pacientes infectados pelo HIV-1 como em voluntários não infectados pelo HIV.
2. A proporção de soroconversão pela vacina contra o H1N1 na dose de 3,75 µg em pacientes infectados pelo HIV-1 é similar à proporção de soroconversão induzida pela mesma vacina na dose de 7,5 µg
3. A proporção de soroconversão com uma dose da vacina contra o H1N1 é semelhante à proporção após a segunda dose.
4. A proporção de soroconversão pela vacina contra o H1N1 não é influenciada pela administração concomitante ou após 21 dias da vacina contra influenza sazonal.

1.2 Objetivos primários

1.2.1 Determinar o desenvolvimento, em cada estrato de contagem de células CD4+, de títulos de anticorpos protetores contra o H1N1 após a série vacinal da vacina contra o H1N1

1.2.2 Avaliar a segurança e a tolerabilidade da vacina contra o H1N1 nas diferentes doses e esquemas de administração

1.3 Objetivos secundários

1.3.1 Avaliar a persistência dos títulos de anticorpos contra o H1N1 após a série vacinal

1.3.2 Avaliar as alterações da carga viral de HIV-1 e da contagem de células CD4+ após a série vacinal.

1.3.3 Avaliar o efeito do nadir e da contagem inicial de células CD4+ nas respostas de anticorpos.

1.3.5 Avaliar os efeitos da carga viral inicial de HIV-1 na resposta de anticorpos.

1.3.4 Determinar as respostas imunológicas celulares e suas correlações ao desenvolvimento e à amplitude das respostas ao H1N1 e comparar as respostas imunológicas celulares.

1.3.5 Avaliar os efeitos da vacinação nos níveis de CD4 e carga viral do HIV

1.3.7 Avaliar os efeitos de HAART na resposta imune à vacina.

1.3.8 Avaliar os efeitos da vacina contra influenza sazonal na tolerabilidade e na resposta imune à vacina anti-H1N1

1.3.9 Determinar as respostas imunológicas celulares e suas correlações ao desenvolvimento e à amplitude das respostas ao H1N1 e comparar as respostas imunológicas celulares.

1.3.10 Avaliar o efeito de imunidade pré-existente para H1N1 nas respostas sorológicas a essa vacina

1.3.11 Avaliar o numero de casos de síndrome gripal influenza like entre os pacientes após a vacinação inicial

1.3.12 Caracterizar os tipos de influenza causando síndrome gripal influenza like na coorte em estudo

1.3.13 Analisar a custo-efetividade das diferentes estratégias de vacinação

2.0 INTRODUÇÃO

2.1 Contexto

A natureza das pandemias de influenza

A disseminação dos vírus de influenza A de origem animal em populações humanas [incluindo os subtipos A/H5N1, H7N7, H9N2 e, mais recentemente, o vírus da influenza H1N1 de origem suína (S-OIV), também designado "Influenza A de 2009 (H1N1)" neste texto] adicionou urgência aos esforços em andamento para desenvolver planos de resposta a potenciais pandemias de influenza (Subbarao et al, 1998; Peiris et al, 1999; Dawood et al, 2009; Peiris et al, 2009).

Houve três pandemias no século passado. Durante a pandemia de influenza A/H1N1 em 1918, estima-se que 40 milhões de mortes ocorreram em todo o mundo (Oxford, 2000; Gatherer, 2009). Foram observados excesso de mortalidade, morbidade e perturbações sociais durante as pandemias de influenza A/H2N2 em 1957 e de influenza A/H3N2 em 1968 (Patriarca & Cox, 1997). Em ambas as ocasiões as populações humanas não tinham níveis significativos de imunidade preexistente para o vírus da pandemia, resultando na rápida propagação dos subtipos de vírus da influenza A. Assim, o surgimento de um novo subtipo de influenza na população humana tem o potencial de promover um quadro de emergência na saúde pública. A influenza A de 2009 (H1N1) atingiu níveis de pandemia, tal como definido pela Organização Mundial de Saúde ("um vírus influenza causando surtos sustentados na comunidade em pelo menos dois países, em pelo menos duas regiões da OMS") (World Health Organization, 2009). Modelos animais indicam que o vírus influenza A (H1N1) de 2009 se replica melhor no pulmão e no trato gastrointestinal em comparação ao H1N1 sazonal, sugerindo que o vírus pandêmico possa causar maior morbidade que o vírus sazonal. A virulência da cepa pandêmica em humanos é ainda incerta.

Os preceitos fundamentais para o preparo para uma pandemia de influenza incluem o reforço da vigilância para a identificação de vírus emergentes, a capacidade ampliada de produzir e entregar as vacinas pertinentes, a disponibilidade de antivirais para a prevenção e o tratamento das infecções causadas por vírus pandêmico, além de infra-estrutura de saúde pública adequada para gerir e coordenar os esforços de controle.

As ameaças de pandemia de influenza que ocorreram em 1976 (gripe suína) e em 1977 (gripe russa) resultaram em um programa de desenvolvimento de

vacina sem adjuvantes e inativada contra o vírus influenza, que trouxe importantes informações sobre as variáveis que influenciam a resposta imunológica à imunização. (Oxford, 2000; Gatherer, 2009). Os principais avanços observados foram sobre dosagens de hemaglutinina (HA) na vacina, número de doses necessárias (1 ou 2) e o tipo de vacina administrada (vírus inteiro, partes do vírus ou antígeno de superfície purificado). Os estudos com a vacina contra a influenza demonstraram ainda que os fatores específicos do hospedeiro que influenciam as respostas imunológicas são idade, pré-ativação, presença de doença subjacente e uso de medicamentos imunossupressores.

Resposta imunológica à vacina contra a influenza

A hemaglutinina (HA) é o receptor viral que se liga aos receptores das células epiteliais do hospedeiro, permitindo que o vírus penetre na célula hospedeira. Portanto, os anticorpos contra a HA desempenham um papel importante na imunidade que protege da infecção pelo vírus influenza e são a base para o licenciamento de vacinas contra a influenza. A resistência à infecção por cepas de vírus da influenza sazonal correlaciona-se diretamente tanto com a inibição da hemaglutinação no soro (*hemagglutination inhibition* - HAI) como com os níveis de anticorpos de neutralização (Nt) Medidas séricas de HAI e anticorpos NT são utilizadas para avaliar a imunogenicidade das vacinas contra a influenza sazonal e pandêmica (Couch & Kasel, 1983; Ennis et al, 1977).

A magnitude da resposta imunológica à vacina inativada sem adjuvantes contra a influenza é grandemente afetada pela dosagem dos antígenos (geralmente expressa em peso de HA) contidos na vacina. Estudos avaliando o efeito da dose de HA nas respostas imunológicas às vacinas inativadas sem adjuvantes contra a influenza sazonal realizados nos últimos 35 anos demonstraram aumentos relacionados com a dose do soro e das respostas de anticorpos nas mucosas (Ennis al, 1977; Ruben &, Jackson, 1972; Ruben et al, 1975; Matzkin & Nili, 1984; Palache et al, 1993; Gross et al, 1988; Keitel et al, 1994; Keitel et al, 1996; Keitel et al, 2008).

Doses mais altas da vacina estão também associadas com o desenvolvimento de níveis mais altos de anticorpos séricos que reconhecem variantes derivadas antigenicamente distintas e podem superar as respostas subideais em vacinados imunologicamente expostos, como os pacientes idosos (Keitel et al . 2006; Falsey et al, 2009).

Foi avaliado o efeito da dose de HA na imunogenicidade de vacinas inativadas cujos alvos são algumas cepas de potenciais pandemias de vírus influenza (H9N2, H5N1) (Atmar et al, 2006; Treanor JJ, Wilkinson BE, Masseoud et al, 2001; Treanor et al, 2006). Embora doses mais altas de HA induzam de rotina uma resposta significativamente maior do que uma única dose, a resposta ainda pode ser insuficiente se o antígeno não for altamente imunogênico. Por exemplo, as vacinas contra o H5N1 são muito menos imunogênicas do que as vacinas sazonais ou vacinas contra o H9N2. Duas doses de 90 mcg de qualquer baculovírus recombinante expressando HA H5N1 ou de uma vacina inativada de antígeno de subvírion promoveram resposta de anticorpos em apenas cerca de 50% dos jovens adultos saudáveis (Atmar et al, 2006; Treanor

et al, 2001). Doses mais altas de HA também estão associados a eventos adversos mais freqüentes.

A via de administração pode afetar a resposta imunológica. A administração intranasal de vacinas de vírus vivo atenuado ou virossomal foi proposta para melhorar as respostas locais, inclusive nos pacientes infectados pelo HIV-1 (Hammitt et.al, 2008; Quan et al, 2008). A administração intradérmica de vacinas inativas sem adjuvantes contra a influenza gera títulos mais elevados de anticorpos em comparação à administração intramuscular das mesmas preparações contendo HA (Gelinck et al, 2009; Holland et al, 2008; Leroux-Roels et al, 2008)

O uso de adjuvantes é outra abordagem para melhorar a imunogenicidade das vacinas contra a influenza (Galli et al, 2009; Vesikari et al, 2009) Adjuvantes têm o potencial de melhorar as respostas imunológicas séricas a uma determinada dose de antígeno, diminuir a quantidade de antígeno necessária na vacina (poupando doses) e melhorar as respostas imunológicas de grupos que geralmente respondem mal aos antígenos inativados (por exemplo, imunodeprimidos, idosos) (Atmar et.al, 2009.). Novos e mais potentes adjuvantes não foram avaliados em pacientes infectados pelo HIV.

Nova cepa pandêmica de influenza

Recentemente, um novo vírus de influenza de origem suína, o A/H1N1, agora designada Influenza A (H1N1) de 2009, foi identificado como uma importante causa de doenças respiratórias febris no México e nos Estados Unidos (EUA) (Dawood et al, 2009; Peiris et al, 2009; Gatherer 2009)

Em um relatório recente sobre doença respiratória grave concomitante à circulação da Influenza A (H1N1) de 2009, no México, descreve-se que foram afetadas pessoas de todas as idades. No entanto, em comparação com dados de épocas anteriores de influenza, ocorreu maior aumento da mortalidade em crianças e adultos jovens. A Influenza A (H1N1) de 2009 rapidamente se espalhou para vários países ao redor do mundo, fazendo com que a Organização Mundial de Saúde declarasse a pandemia em 11 de junho de 2009 (World Health Organization. http://www.carec.org/influenzaa-H1N1-pandemic-declaration.html. 2009.8). Dados sorológicos de várias coortes em diferentes faixas etárias que receberam vacina contra a influenza sazonal trivalente licenciada (TIV) sugerem que essa vacina provavelmente não proporciona proteção contra o novo vírus (Centers for Disease Control and Prevention, 2009). Observou-se ainda que cerca de 33% dos indivíduos com mais de 60 anos de idade têm níveis mensuráveis de anticorpos séricos HAI ou Nt contra o Influenza A (H1N1) de 2009, enquanto aos jovens adultos e crianças faltam completamente títulos protetores. Esses dados indicam a necessidade de desenvolver vacinas contra a nova cepa de Influenza A (H1N1) de 2009 que traga proteção conveniente para pessoas de diferentes faixas etárias; especialmente para aqueles com um ou mais fatores de risco para doença grave.

Influenza nos pacientes infectados pelo HIV-1

Os pacientes infectados pelo HIV-1 não apenas sofrem a morbidade típica da influenza sazonal e a potencial interferência da doença na resposta à terapia antirretroviral, mas também a possibilidade de a influenza ser mais grave que nas pessoas não infectadas da mesma faixa etária (Fine et al, 2001; Madhi et al, 2002).

Embora até hoje os trabalhos sugiram que as pessoas saudáveis têm tipicamente doença leve após infecção pelo vírus H1N1, doenças subjacentes, incluindo a imunodeficiência, parecem aumentar o risco de doença grave e até a morte com durante a pandemia atual de influenza. (Hackett et al, 2009).

Os pacientes infectados pelo HIV-1 também podem eliminar vírus influenza por períodos maiores de tempo e apresentar infectividade continuada, necessitando de isolamento na clínica ou no hospital. (Mendoza Sanchez et al, 2006)

Resposta à vacina contra a influenza sazonal em pacientes infectados pelo HIV-1

Em pacientes que não têm doença progressiva pelo HIV-1 e/ou estão recebendo TARV altamente ativa (HAART), estas respostas são melhores (King et al, 2000; Levin et al, 2008; Vigano et al, 2008).

Os estudos que avaliaram a segurança e a eficácia da vacina contra influenza sazonal mostram que a administração do produto é segura nessa população, porém os resultados de eficácia são contraditórios. Vários estudos clínicos mostraram que as respostas de anticorpos a TIV são diminuídas em crianças e adultos infectados pelo HIV-1 (Staprans et al, 1995; Amendola et al, 2001; Zanetti et al, 2002; Brydak et al, 1999; Kroon et al, 1998; Nelson et al, 1988; Miotti et al, 1989; Kroon et al, 1998), especialmente nos indivíduos que não estavam em uso de terapia antirretrovial (Kroon et al, 1994; Miotti et al,1989; Chadwick et al, 1994; Fowke et al, 1997; Nelson et al, 1988; Kroon et al, 1998; Brydak et al, 1999)), porém outros estudos não obtiveram os mesmos resultados (Tasker et al, 1999; Amendola et al, 2001; Zanetti et al, 2002). Dois estudos recentes mostraram que a resposta humoral à vacina é relacionada aos níveis de RNA viral de forma mais significativa que à contagem de CD4 em sangue periférico, no momento da vacinação (Evison et al, 2009; Yamanaka et al, 2005). Em estudos que utilizaram como desfecho a proteção clínica conferida pela vacina observou-se que portadores do HIV apresentavam, após a vacinação, menos casos e quadros menos graves de influenza quando comparados aos não vacinados Yamanaka et al, 2005; Fine et al, 2001; Ranieri et al, 2005). O único estudo sobre a vacina contra influenza sazonal que foi randomizado, duplo-cego e controlado por placebo mostrou uma redução absoluta de 20% do risco de sintomas respiratórios (de 49% para 29%, p = 0,04) e 100% de proteção (IC 95% 73 a 100%) contra a influenza sintomática confirmada laboratorialmente (p < 0,001) entre os pacientes infectados com

HIV que recebem a vacina da influenza em comparação com aqueles que recebem placebo (Tasker et al, 1999). Duas revisões sistemáticas baseadas nos quatro estudos de desfechos clínicos citados anteriormente concluíram que a vacinação contra a influenza de adultos infectados pelo HIV pode ser eficaz, apesar das respostas variáveis de anticorpos (Atashili et al, 2006; Anema et al, 2008). As duas revisões destacam a limitação dos dados disponíveis e a necessidade de mais estudos maiores randomizados (apenas um foi realizado até hoje) para confirmar a eficácia e a segurança da vacinação nesta grande população de pacientes, particularmente entre aqueles com contagem de CD4+ muito baixa.

Respostas vacinais em pacientes com HIV/AIDS

À medida que a disponibilidade de esquemas anti-retrovirais potentes aumentou a expectativa e a qualidade de vida dos pacientes infectados pelo HIV, a resposta às vacinas contra as infecções que podem ser prevenidas adquiriram maior significado. Respostas insuficientes após vacinação contra hepatite A e hepatite B foram observadas e documentadas em pacientes infectados pelo HIV (Bruguera et al, 1992; Keet et al, 1992;Rimland & Guest, 2005; Shire et al, 2006; Tayal & Sankar 1994).

Uma meta análise de oito estudos desenhados para avaliar a eficácia da vacinação contra a hepatite A entre pacientes infectados pelo HIV utilizando a série vacinal convencional revelou um baixo índice de resposta. (Shire et al, 2006). O índice geral de resposta para os pacientes infectados pelo HIV foi de 64%, observado em uma associação total de 458 participantes. Cada estudo revelou uma baixa proporção de pacientes obtendo títulos adequados de anticorpos contra o HAV. Em outro estudo, uma análise retrospectiva mostrou que o índice de resposta à vacina contra a hepatite A foi de apenas 48% em comparação aos índices descritos de 100% entre pacientes HIV-negativos. (Weissman et al, 2006).

Comparado ao sucesso dos índices de soroconversão acima de 90% observados em hospedeiros imunocompetentes, os pacientes infectados pelo HIV respondem à vacina contra o HBV com índices de 17,5% a 56%. (Bruguera et al, 1992; Keet & van Doornum, 1992; Tayal & Sankar, 1994; Overton et al, 2005).

As estratégias para melhorar os índices de resposta à vacina compreendem o uso de doses maiores, a administração de maior número de doses e a administração de reforços vacinais. (Bruguera et al, 1990; Idilman et al, 2003). Doses mais altas de vacina inativada contra o HBV têm demonstrado melhor resposta de anticorpos em jovens infectados pelo HIV (Flynn et al, 2009).

Na população infectada pelo HIV existem indícios sugerindo que uma resposta adequada de anticorpos possa não se manter ao longo do tempo, como esperado. O uso de uma estratégia de seis doses promovendo títulos de anticorpos iniciais comparáveis aos da população imunocompetente observou que títulos adequados contra o HBV só se mantiveram em 59% dos

participantes um ano após o término da série modificada (Rey et al, 2000).

Os pesquisadores analisaram os indicadores específicos do paciente associados a resposta à vacina contra a hepatite A e a hepatite B. A presença de baixos níveis de viremia do HIV foi sistematicamente associada à falha na resposta à vacinação contra hepatite. Em um modelo de regressão logística, Overton mostrou que apenas níveis de RNA do HIV abaixo de 400 cópias/mL no momento da vacinação contra a hepatite foram associados a resposta protetora, enquanto a contagem de CD4+ no momento da vacinação não foi considerada estatisticamente significativa (Overton et al, 2005). Em crianças infectadas pelo HIV níveis indetectáveis de carga viral do HIV e contagem percentual de células T CD4+ indicaram resposta à vacina contra hepatite A (Weinberg et al, 2006). O nadir da contagem de CD4+ não foi associado à previsão da produção de anticorpos protetores nos pacientes com HIV nem para a vacina contra a hepatite A, nem para a vacina contra a hepatite B em nenhum estudo até hoje.

2.2 Justificativa

As seguintes considerações serviram de base para que esse estudo fosse proposto:

1. A infecção pelo HIV aumenta o risco de quadros mais graves e de complicações da infecção por influenza, especialmente as infecções bacterianas secundárias;

2. Estudos com outras vacinas, tais como do vírus da hepatite B (HBV), sugeriram que o aumento do título de antígenos da vacina aumenta a resposta de anticorpos nas pessoas infectadas pelo HIV;

3-. Dados de outras populações sugerem maior resposta de anticorpos a doses mais altas da vacina da influenza sazonal;

4- É muito provável que uma nova onda da epidemia de influenza A (H1N1) de 2010 ocorra no Brasil no outono-inverno de 2010, quando as interações sociais e as condições climáticas tornam-se ainda mais propícias à propagação do vírus da influenza.

Nesse contexto, o conhecimento da segurança e da imunogenicidade da vacina A/H1N1 S-OIV (nome comercial: Pandemrix®), produzida pela Glaxo Smith & Kline (GSK), em adultos infectados pelo HIV é extremamente importante para enfrentar as necessidades de saúde dessa população vulnerável.

Como foi demonstrado que a influenza sazonal causa doença mais grave nas pessoas infectadas pelo HIV em relação às pessoas não infectadas da mesma idade (33;34), é provável que a Influenza A (H1N1) de 2009 resulte em significativa morbidade e possível maior mortalidade nas pessoas infectadas pelo HIV. A morbidade pode ser uma conseqüência direta do vírus da influenza ou a infecção pode resultar em infecções bacterianas secundárias ou

diminuição da adesão à terapia antirretroviral do paciente devido a náuseas e vômitos graves que podem ocorrer como manifestações da influenza. Assim, a prevenção da infecção nesta população é fundamental.

A equipe desse estudo entende que adultos infectados pelo HIV-1 têm maior risco de doenças graves e morte pelo vírus de origem suína da influenza H1N1, já que a infecção pelo HIV aumenta o risco de má resposta a doses de vacinas que são imunogênicas para outras populações. O uso de uma dose mais alta (7,5 µg) da vacina A/H1N1 da GSK neste estudo irá alcançar os objetivos científicos de avaliação oportuna da segurança e da imunogenicidade do produto vacinal nessa população de alto risco, maximizando o potencial de os participantes do estudo serem protegidos pelo estudo da vacina H1N1 de origem suína nesta temporada.

Este estudo irá avaliar a segurança e a imunogenicidade após cada uma das duas doses da vacina A/H1N1 S-OIV da GSK entre adultos infectados pelo HIV-1 num centro de Referencia para pacientes com HIV/AIDS no Rio de Janeiro. Acredita-se serem necessárias duas doses porque a maioria dos participantes do estudo provavelmente não teve exposição anterior à Influenza A (H1N1) de 2009. Como a vacina da influenza sazonal resulta muitas vezes em menor resposta entre as pessoas infectadas pelo HIV-1, optamos por investigar a maior dose de antígeno, 7,5 µg, em comparação com a dose de 3,75 µg que está sendo atualmente utilizada. Também optamos por estratificar a nossa população de estudo em 2 grupos com base na contagem de linfócitos CD4. Os grupos foram selecionados para fornecer informações de resposta à vacina e de segurança nas diferentes faixas da imunodeficiência.

A fim de compreender o mecanismo da doença e da proteção conferida pelos diferentes esquemas vacinais, vamos investigar a resposta sérica (desenvolvimento de anticorpos específicos em níveis protetores), o tempo de duração da resposta humoral e o desenvolvimento de síndrome gripal causada pelo H1N1 após a vacinação desta população.

Propomos também a investigação da resposta celular à vacina. A geração de respostas dos linfócitos T citotóxicos (CTL) contra a Influenza A (H1N1) de 2009 é de particular interesse, porque o vírus se replica melhor no tecido pulmonar que a influenza sazonal e as CTL são o principal mediador da eliminação viral nos pulmões. As células de memória B para a Influenza A (H1N1) de 2009 irão assegurar que o hospedeiro responda adequadamente à exposição ao vírus selvagem.

Em resumo, a Influenza A (H1N1) de 2010 tem probabilidade de infectar uma proporção significativa de adultos infectados pelo HIV-1 se não houver uma vacina eficaz disponível antes da infecção se espalhar. A infecção irá provavelmente causar doença grave nessa população vulnerável, portanto, os esforços para determinação do melhor esquema vacinal são críticos. A imunogenicidade da vacina candidata contra a Influenza A (H1N1) de 2009 deve ser comprovada nos adultos infectadas HIV-1 de modo a assegurar que esta população está protegida. A falta de uma resposta imunológica protetora corrobora a necessidade de medidas adicionais para proteger esta população

de alto risco.

Fundamentos em termos de imunogenicidade

As pessoas infectadas pelo HIV sabidamente apresentam pior resposta às séries vacinais convencionais como as da hepatite A e B em comparação às pessoas sem infecção pelo HIV. Os pesquisadores analisaram indicadores específicos de pacientes associados a baixa resposta e observaram que baixa contagem de células CD4 e carga viral detectável do HIV estavam associados a baixa resposta à vacinação contra hepatite A e B em alguns estudos. Neste estudo, iremos avaliar a segurança e a imunogenicidade da vacina contra o vírus influenza A H1N1. Este estudo será estratificado pela contagem de células CD4 para avaliar se este fator interfere ou não na capacidade do participante de fabricar anticorpos.

A imunogenicidade será medida através de testes sorológicos de anticorpos anti influenza H1N1. Os métodos para determinação dos limiares do *status* sorológico estão em fase de seleção e de padronização pelo Laboratório de Vírus Respiratório e do Sarampo do IOC. Tão logo os métodos a serem usados form definidos será escrita uma emenda a este protocolo, detalhando o que será feito.

As análises serão conduzidas em pelo menos nove momentos ao longo do estudo, previamente à administração da dose vacinal inicial (Dia zero), imediatamente antes da administração da segunda dose vacinal e/ou vacina contra influenza sazonal (Dia 21), vinte e um dias a administração da segunda dose da vacina e/ou da vacina contra influenza sazonal (Dia 42) e nos seguintes pontos após esquema vacinal completo contra H1N1: 8, 16, 24, 32, 40 e 48 semanas. Nos voluntários com infecção pelo HIV, além da avaliação nestes momentos será feita tmbém sorologia nas semanas 8, 16, 24, 32, 40 e 48 após a primeira dose da vacina.

A imunogenicidade também será determinada pela avaliação das respostas imunológicas celulares à vacinação contra o H1N1 e suas correlações ao desenvolvimento e à amplitude das respostas ao H1N1. A determinação das respostas em cada estrato de contagem de células CD4+ irá permitir comparações mais rigorosas entre os estratos. Quanto aos indivíduos infectados pelo HIV-1, para cada estratificação de CD4 definida no estudo e para cada esquema vacinal a ser aplicado, serão selecionados dez pacientes, de acordo com a disponibilidade de células obtidas do sangue periférico, para a avaliação da resposta imune celular, através do método de ELISpot, totalizando 40 indivíduos. Para a mesma avaliação, dez voluntários do grupo controle serão aleatoriamente selecionados. As análises serão conduzidas em pelo menos cinco momentos ao longo do estudo, previamente à administração da dose vacinal inicial (Dia zero), imediatamente antes da administração da segunda dose vacinal e/ou vacina contra influenza sazonal (Dia 21), vinte e um dias após a administração da segunda dose da vacina e/ou da vacina contra influenza sazonal: 21 dias (Dia 42) e nos seguintes momentos após o esquema vacinal completo contra influenza H1N1, 24 e 48 semanas. O ELISpot será realizado no Laboratório de AIDS e Biologia Molecular do IOC.

3.0 DESENHO DO ESTUDO

Estudo de fase II, aberto e de não-inferioridade, para avaliar a imunogenicidade, reatogenicidade, segurança e tolerabilidade de dois esquemas vacinais contra o H1N1 em pacientes adultos infectados pelo HIV, em comparação com o esquema vacinal clássico (dose única) em indivíduos HIV negativos. Nos voluntários infectados pelo HIV a vacina será administrada nos dias zero e 21 e os participantes serão acompanhados durante 48 semanas após a última dose.

Pacientes infectados pelo HIV que preencham os critérios de inclusão para o estudo serão aleatoriamente selecionados para participar do estudo e posteriormente randomizados para receber um de dois esquemas vacinais:
- Esquema 1: Aplicações de 3,75 µg da vacina (uma dose via IM) nos dias 0 e 21
- Esquema 2: Aplicações de 7,5 µg da vacina (duas doses via IM) nos dias 0 e 21

Os voluntários do grupo controle receberão uma aplicação única de 3,75 µg da vacina, via IM.

O estudo irá selecionar 180 pacientes infectados pelo HIV-1 em cada um dos dois estratos de contagem de células CD4, posteriormente randomizados para receber um dos dois esquemas vacinais do estudo, totalizando 360 pacientes HIV positivos incluídos para análise. Os dois estratos de CD4 serão abertos simultaneamente para inclusão.

Estrato A (n = 180): contagem de células CD4 > 200 células/mm^3. - 90 voluntários desse estrato receberão o esquema vacinal 1 e outros 90 o esquema vacinal 2. Os participantes serão selecionados aleatoriamente para receber um dos dois esquemas vacinais.

Estrato B (n = 180): contagem de células CD4 ≤ 200 células/mm^3 - 90 voluntários desse estrato receberão o esquema vacinal 1 e outros 90 o esquema vacinal 2. Os participantes serão selecionados aleatoriamente para receber um dos dois esquemas.

Serão recrutados 90 indivíduos sem infecção pelo HIV, que receberão o esquema vacinal clássico (3,75 µg em dose única).

Adicionalmente, todos os participantes do estudo receberão vacina contra influenza sazonal, em dose única, 21 dias após a primeira dose (ou a dose única, para o grupo controle) da vacina contra H1N1.

Os 10 primeiros voluntários incluídos em cada um dos cinco sub-grupos (dois estratos de CD4, dois diferentes esquemas vacinais e indivíduos HIV negativos) colherão sangue para a avaliação de resposta celular à vacina.

4.0 ESCOLHA E RECRUTAMENTO DOS PARTICIPANTES

4.1 Critérios de inclusão

Serão incluídos no estudo 360 pacientes infectados pelo HIV e 90 voluntários saudáveis, ambos os grupos com idade entre 18 e 59 anos. Esse estudo só será iniciado após aprovação, pelo Comitê de Ética em Pesquisa do IPEC, desse protocolo, assim como de todo material de divulgação a ser utilizado para o recrutamento de sujeitos.

Critérios de inclusão para todos os participantes

A1. Ambos os sexos, com idade entre 18 e 59 anos;

A2. Capacidade de dar consentimento voluntariamente para a participação no estudo;

A3. As participantes do estudo com potencial de reproduzir (definido como meninas após a menarca ou mulheres que não chegaram à menopausa pelo menos durante 24 meses consecutivos, ou seja, que menstruaram nos últimos 24 meses, ou não foram submetidas a esterilização cirúrgica) precisam ter resultado negativo ao exame de gravidez, sérico ou urinário, no momento da triagem para o estudo e nos dias de aplicação de vacina. Caso pratiquem atividades sexuais passíveis de promover gestação, as participantes precisam usar algum tipo de contracepção durante o período do estudo. Pelo menos um dos seguintes métodos TEM DE ser usado de modo adequado:

- Preservativos (masculinos ou femininos)
- Anticoncepcionais hormonais

As participantes do estudo sem potencial de reproduzir [definido como meninas antes da menarca ou mulheres após a menopausa pelo menos durante 24 meses consecutivos ou mulheres submetidas a esterilização cirúrgica (p. ex., laqueadura tubária bilateral, procedimento Essure® ou qualquer procedimento contraceptivo permanente não-incisional, ooforectomia bilateral ou salpingectomia bilateral)] são elegíveis sem exigência de uso de contracepção. O relato da participante constitui documento aceitável de esterilização, menopausa e potencial reprodutivo das mulheres.

A4. Resultados laboratoriais no momento da triagem para o estudo:

- Hemoglobina > 8,0 g/dL;
- Bilirrubina direta < 2,5 x o limite normal superior;
- Aminotransferase alanina, ALT (TGP) e aminotransferase aspartato, AST (TGO) < 3 x o limite superior de normalidade;
- Contagem de plaquetas $\geq$ 100.000/mm^3

Critérios de inclusão exclusivos para os participantes com infecção pelo HIV:

B1. Infecção pelo HIV-1 (comprovada por teste rápido do HIV ou kit de ELISA aprovado e confirmado pela repetição do ELISA, IFI, *Western blot, carga* viral plasmática do HIV-1) a qualquer momento antes do ingresso no estudo.

> A confirmação do resultado do teste inicial deve usar um teste diferente do utilizado para a avaliação inicial. Um teste rápido inicial reagente deve ser confirmado por outro teste rápido ou por um teste ELISA baseado em uma diferente preparação de antígenos e/ou teste com diferente princípio (p.ex., indireto *vs*. competitivo), ou um *Western blot* ou carga viral plasmática do HIV-1. Um resultado inicial de ELISA tem de ser confirmado por outro método como teste rápido, *Western blot* ou carga viral plasmática de HIV-1, mas não pela repetição do próprio ELISA

B2. Sem modificação da situação de tratamento antirretroviral nas 8 semanas anteriores ao momento da triagem para o estudo:

- Se o voluntário estiver em uso de HAART é necessário que esteja usando o mesmo esquema durante pelo menos 8 semanas antes da triagem. Modificações de posologia dos anti-retrovirais nas 8 semanas anteriores ao ingresso no estudo são permitidas. Além disso, é permitida a troca de apresentação farmacológica (p. ex., da apresentação convencional para apresentação em associações).
- Se o voluntário não estiver em tratamento antirretroviral regular não deve ter recebido nenhuma dose de nenhum antirretroviral nas 8 semanas que antecedem a triagem, incluindo profilaxia de transmissão vertical do HIV (esquemas profiláticos e terapêuticos prévios são permitidos)

B3. Sem planejamento de trocar ou iniciar HAART nos próximos 6 meses.

Critérios de inclusão exclusivos para os participantes sem infecção pelo HIV (grupo controle):

C1 Sorologia anti-HIV negativa comprovada por teste rápido aprovado no Brasil

C2. Profissionais de saúde com indicação de receber vacina contra H1N1

4.2 Critérios de exclusão

Os critérios de exclusão descritos a seguir serão usados para selecionar tanto os portadores do HIV quanto os voluntários não infectados:

D1. Uso de qualquer tratamento sistêmico antineoplásico ou imunomodulador, corticóide sistêmico, vacinas experimentais, interleucinas, interferons, fatores

do crescimento ou imunoglobulina intravenosa (IVIG) nos 45 dias que antecedem o ingresso no estudo.

D2. Gestação ou aleitamento.

D3. Alergia e/ou sensibilidade ou qualquer hipersensibilidade conhecida aos resíduos presentes na vacina (ovo, proteína de galinha, ovalbumina, formaldeído, sulfato de gentamicina e deoxicolato de sódio) e/ou ao tiomersal.

D4. Uso de álcool ou dependência ou outros quadros que, na opinião do pesquisador do centro, possam interferir no cumprimento das exigências do estudo.

D5. Doença grave exigindo tratamento sistêmico e/ou hospitalização nos 45 dias que antecedem o ingresso no estudo

D6. Doença febril grave ou infecção aguda no momento da triagem para o estudo e/ou no dia de aplicação da vacina

D6. Vacinação contra influenza sazonal nos últimos 12 meses antes da entrada no estudo

D7. Vacinação contra influenza A H1N1 previamente

D8. História ou antecedente familiar de síndrome de Guillain-Barré (pais, irmãos, meio-irmãos ou filhos).

D9. Diagnóstico de quadro neurológico incluindo (mas não se limitando a) ausência de reflexos profundos calcâneos e patelares em ambas as pernas (os quatro ausentes) nos últimos seis meses.

D10. Perda de força desproporcional no(s) membro(s) inferior(es) em comparação aos membros superiores nos últimos seis meses.

4.3 Procedimentos de recrutamento no estudo

Os voluntários serão recrutados dentre os indivíduos que procurarem o IPEC para receber vacina contra a influenza AH1N1 e na coorte de pacientes com infecção pelo HIV do IPEC, de acordo com os grupos prioritários a serem vacinados definidos pelo Ministério da Saúde do Brasil.

Este estudo só será iniciado após aprovação do protocolo e do termo de consentimento livre e esclarecido pelo Comitê de Ética em Pesquisa (CEP) do IPEC. Qualquer material que venha a ser divulgado para ajudar no recrutamento de voluntários será previamente aprovado pelo CEP do IPEC.

Quando um candidato a participar do estudo for identificado, os detalhes serão cuidadosamente discutidos com o mesmo. O participante será solicitado a ler e assinar o termo de consentimento livre e esclarecido aprovado do protocolo antes que qualquer procedimento do estudo seja realizado.

5.0 TRATAMENTO DO ESTUDO

O tratamento do estudo é definido como sendo a vacina A/H1N1 S-OIV, produzida pela GSK e será fornecido pela equipe do estudo aos voluntários. A equipe do estudo também fornecerá a vacina contra influenza sazonal.

Os pesquisadores negociaram com o Ministério da Saúde brasileiro o fornecimento de vacinas do mesmo fabricante em quantidade suficiente para todos os voluntários incluídos.

5.1 Esquemas e administração da vacina.

5.1.1 Esquemas

Os participantes do estudo irão receber a vacina contra o H1N1 por via intramuscular (IM) na região deltóide do membro superior, em um de três diferentes esquemas:

- Duas aplicações de uma dose (3,75 mcg) cada, nos dias zero e 21 (dois estratos de contagem de CD4 para infectados pelo HIV).
- Duas aplicações de duas doses (7,5 mcg) cada nos dias zero e 21 (dois estratos de contagem de CD4 para infectados pelo HIV).
- Uma aplicação de uma dose (3,75 mcg) única (grupo controle)

Adicionalmente, dose única da vacina contra influenza sazonal será aplicada no dia 21 para os todos os grupos de voluntários.

5.1.2 Administração da vacina

A vacina contra o H1N1 será administrada de acordo com o cronograma de visitas do estudo para cada grupo avaliado.

Antes da administração o produto deve ser reconstituído de acordo com as orientações do fabricante, detalhadas no item 5.2 deste protocolo.

A vacina será administrada por via intramuscular, preferencialmente na região deltóide ou na região antero-lateral da coxa. A equipe do estudo usará técnicas adequadas de assepsia durante o preparo e a administração da vacina. Todas as injeções devem ser aplicadas preferencialmente no membro não-dominante; entretanto, se isso não for possível, pode-se aplicar no dominante.

A aplicação da vacina não pode ser intravascular, subcutânea ou intradérmica.

5.2 Apresentação do produto do estudo e seu preparo

O produto Pandemrix® é fornecido em dois recipientes, a suspensão (frasco de 2,5ml, com tampa de borracha, contendo o antígeno) e a emulsão (frasco de 2,5 ml, com tampa de borracha, contendo o adjuvante), ambos multidose. Uma

embalagem do produto contém 50 frascos de suspensão e 50 frascos de emulsão. O produto deve ser conservado entre 2 e 8°C e deve ser mantido na embalagem original, protegido contra a luz.

O produto deve ser reconstituído imediatamente antes do uso, respeitando-se os seguintes procedimentos:

- Tirar um frasco de suspensão e um frasco de emulsão da geladeira e aguardar até atingirem a temperatura ambiente;
- Agitar e inspecionar visualmente cada frasco – caso se verifique a presença de qualquer partícula estranha o produto não deverá ser aplicado;
- Aspirar o conteúdo do frasco de emulsão com seringa estéril e injetar no frasco de suspensão;
- Agitar bem o frasco reconstituído (o produto deverá ter aspecto de um líquido turvo esbranquiçado, sem partículas, com volume de 5ml);
- Aspirar o volume equivalente à dose prescrita para o voluntário usando seringa descartável e injetar imediatamente (cada 0,5ml contém 3,75 μg de antígeno)

Após a reconstituição o produto pode ser conservado em temperatura ambiente (25 °C) ou na geladeira e usado em no máximo 24 horas. Caso tenha ficado na geladeira, o produto deve atingir a temperatura ambiente antes da aplicação. Imediatamente antes de cada aplicação o frasco reconstituído deve ser agitado e inspecionado visualmente.

A vacina contra influenza sazonal deverá ser aplicada no dia 21 para os todos os grupos de voluntários, de acordo com as instruções do fabricante. Nos voluntários com infecção pelo HIV, que receberão as duas vacinas num mesmo dia, os produtos devem ser aplicados em membros diferentes.

Produtos não utilizados devem ser descartados de acordo com as normas de biossegurança do IPEC.

5.3 Farmácia: Fornecimento, distribuição e contabilidade do produto.

O farmacêutico do estudo é responsável pelo recebimento, armazenamento, dispensação e contabilidade de todos os produtos do estudo recebidos (Division of AIDS, 2008).

O farmacêutico do centro deve manter um registro completo de todos os produtos do estudo recebidos, arquivando a nota fiscal ou outro documento que comprove o envio a cada novo recebimento. O farmacêutico deve sempre conferir a quantidade e a integridade dos produtos do estudo recebidos. Qualquer inconsistência deve ser imediatamente comunicada ao fornecedor. A temperatura durante o transporte deve ser monitorada. Os produtos do estudo devem ser transportados a uma faixa de temperatura de 2 a 8ºC.

O farmacêutico deve manter o inventário dos produtos atualizado, utilizando um formulário de contabilidade para registrar cada dispensação do produto em investigação ou a cada novo envio do fornecedor O farmacêutico deve realizar

um inventário físico pelo menos uma vez ao mês, a fim de verificar se a quantidade real corresponde a quantidade documentada.

A dispensação dos produtos do estudo deve ser feita diretamente ao profissional de saúde pertencente a equipe do estudo que irá realizar a administração no paciente. A dispensação só poderá ser feita mediante prescrição médica, assinada por um prescritor autorizado pelo pesquisador responsável. O farmacêutico deve se assegurar de que o termo de consentimento livre e esclarecido foi assinado antes de dispensar os produtos do estudo. Todo procedimento de dispensação deve ser documentado no prontuário do paciente. Serão dispensados produtos suficientes para vacinar grupos de voluntários agendados para um mesmo dia. O produto será dispensado na embalagem original, não reconstituído e será transportado até o local da aplicação em embalagens térmicas contendo gelo.

O farmacêutico deve monitorar a temperatura de armazenamento dos produtos em investigação. As vacinas devem ser armazenadas em refrigerador dedicado somente ao armazenamento de vacinas, a uma faixa de temperatura de 2-8ºC. Deve haver um controle contínuo da temperatura, havendo o registro pelo menos a cada 30 minutos. A temperatura também deve ser checada manualmente duas vezes ao dia como plano back-up. Além disso, um alarme de temperatura deve avisar ao farmacêutico por telefone a cada desvio de temperatura registrada. Em caso de queda de energia, um gerador deve ser acionado. Todos os equipamentos utilizados para o controle de temperatura devem ser calibrados a cada 6 meses.

Apenas o farmacêutico do estudo deve ter acesso aos produtos do estudo. Todos os produtos do estudo que não forem utilizados devem ser devolvidos ou destruídos após o término ou a suspensão do estudo.

5.4 Medicamentos concomitantes

No momento não existem dados sobre a administração concomitante de Pandemrix® com outras vacinas, nem sobre a aplicação em indivíduos que usam outros medicamentos. No intuito de evitar eventos adversos causados por interações medicamentosas os pesquisadores devem consultar as bulas mais recentes da vacina em estudo e dos medicamentos concomitantes sempre que um medicamento concomitante for iniciado ou sua dose modificada.

O centro também precisa consultar a bula mais recente do produto do estudo para ter acesso a informações atuais sobre os medicamentos proibidos ou que exijam precauções.

6.0 AVALIAÇÕES CLÍNICAS E LABORATORIAIS

6.1 Cronograma de eventos

O cronograma de eventos do estudo, a ser seguido para cada voluntário incluído, está descrito no anexo 1 deste protocolo de pesquisa (fluxograma de procedimentos). A descrição detalhada dos procedimentos a serem realizados em cada consulta do estudo está registrada no item 6.2 deste protocolo, assim como a janela permitida entre cada consulta.

As consultas interinas para avaliação clínica e coleta de secreção respiratória na vigência de quadros respiratórios agudos serão realizadas a qualquer momento a partir da primeira dose da vacina (dia zero) e não constam do fluxograma de procedimentos (anexo 1).

6.2 Momentos das avaliações

A seguir estão descritas as consultas do estudo e quais procedimentos devem ser realizados em cada uma delas.

Considerando que os frascos de Pandemrix® contém mais de uma dose individual de vacina e que o produto deve ser aplicado até 24 horas após a reconstituição, deve-se agendar as consultas de vacinação para grupos de voluntários em número suficiente para o maior número possível de um frasco reconstituído sejam usadas, evitando desperdício de vacinas.

6.2.1 Consulta de triagem dos indivíduos selecionados: dias -20 a -1

Os seguintes procedimentos devem ser realizados na consulta de triagem:

- Obtenção de consentimento
- História médica completa
- Exame físico e sinais vitais
- Exames de segurança
- Teste para HIV nos candidatos a participar do grupo controle
- Teste de gravidez (mulheres com potencial reprodutivo)
- CD4 (só para HIV positivos)
- Verificação de elegibilidade (incluindo exame de HIV para grupo de controle)

6.2.2 Vacina anti-H1N1: dia zero

A consulta de dia zero deve ser feita um a 20 dias após a triagem, sem janela permitida. Os seguintes procedimentos devem ser realizados na consulta do dia zero:

- Atualização de história médica
- Exame físico e sinais vitais
- Teste de gravidez (mulheres com potencial reprodutivo)
- Verificação de elegibilidade
- Coleta de sangue, processamento e armazenagem para sorologia (H1N1)
- Coleta de sangue, processamento e armazenagem para ELI-Spot
- Carga viral do HIV

- Alocação em um dos estratos de CD4 (para voluntários infectados pelo HIV)
- Randomização em um dos esquemas vacinais (para voluntários infectados pelo HIV)
- Aplicação da 1ª dose da vacina Pandemrix®
- Avaliação de reatogenicidade (1 hora)
- Registro de telefones para contato 24 horas após a vacinação
- Fornecimento de diário do voluntário

6.2.3 Consulta telefônica: dia 1

A consulta telefônica deverá ser realizada 20 a 30 horas após a vacinação, sem janela permitida. O seguinte procedimento será realizado:

- Avaliação dirigida de eventos adversos locais e sistêmicos (incluindo reatogenicidade)

6.2.4 Consulta telefônica: dia 7

A consulta de dia 7 deve ser realizada 7 dias após a vacinação , idealmente no mesmo dia da semana (por exemplo, na segunda-feira seguinte à dose para os voluntários vacinados numa segunda-feira), sendo permitida uma janela de mais ou menos um dia. O seguinte procedimento será realizado:

- Avaliação dirigida de eventos adversos locais e sistêmicos (incluindo reatogenicidade)

6.2.5 Segunda dose de vacina anti-H1N1 (HIV positivos), 21º dia pós-esquema vacinal completo (HIV negativos) e dia da vacina contra influenza sazonal (todos): dia 21

A consulta de dia 21 deve ser realizada 21 dias após a vacinação, idealmente no mesmo dia da semana (por exemplo, na terceira segunda-feira seguinte à primeira dose para os voluntários inicialmente vacinados numa segunda-feira), sendo permitida uma janela de 4 dias a mais e nenhum dia a menos (por exemplo, no mínimo no 21º dia e no máximo no 25º dia após a primeira dose). Os seguintes procedimentos devem ser realizados nessa consulta:

- Atualização de história médica
- Exame físico dirigido e sinais vitais
- Avaliação de reatogenicidade (1 hora)
- Captura e registro de eventos adversos
- Captura e registro de medicações concomitantes
- Exames de segurança
- Teste de gravidez (mulheres com potencial reprodutivo)
- CD4 e CV (para voluntários infectados pelo HIV)
- Coleta de sangue, processamento e armazenagem para sorologia (H1N1)
- Coleta de sangue, processamento e armazenagem para ELI-Spot
- Aplicação da 2ª. Dose da vacina Pandemrix® (para voluntários infectados pelo HIV)
- Vacina contra influenza sazonal
- Avaliação de reatogenicidade (1 hora)
- Recolhimento e verificação do diário do voluntário
- Registro de telefones para contato 24 horas após a vacinação

- Fornecimento de diário do voluntário

6.2.6 Consulta telefônica: dia 22
A consulta telefônica deverá ser realizada 20 a 30 horas após a vacinação, sem janela permitida. O seguinte procedimento será realizado:

- Avaliação dirigida de eventos adversos locais e sistêmicos (incluindo reatogenicidade)

6.2.7 Consulta telefônca: dia 27
A consulta de dia 27 deve ser realizada 7 dias após a vacinação, idealmente no mesmo dia da semana (por exemplo, na segunda-feira seguinte à dose para os voluntários vacinados numa segunda-feira), sendo permitida uma janela de 1 dia. O seguinte procedimento será realizado:

- Avaliação dirigida de eventos adversos locais e sistêmicos (incluindo reatogenicidade)

6.2.8 21º dia pós-esquema vacinal completo (HIV positivos) e 21º dia pós vacina contra influenza sazonal (todos): dia 42
A consulta de dia 42 deve ser realizada 21 dias após a vacinação, idealmente no mesmo dia da semana (por exemplo, na terceira segunda-feira seguinte à primeira dose para os voluntários inicialmente vacinados numa segunda-feira), sendo permitida uma janela de mais ou menos 4 dias (por exemplo, no mínimo no 18º dia e no máximo no 25º dia após a dose). Os seguintes procedimentos devem ser realizados nessa consulta:

- Atualização de história médica
- Exame dirigido físico e sinais vitais
- Captura e registro de eventos adversos
- Captura e registro de medicações concomitantes
- Recolhimento do diário do voluntário
- Exames de segurança
- CD4 e CV (para voluntários infectados pelo HIV)
- Coleta de sangue, processamento e armazenagem para sorologia (H1N1)
- Coleta de sangue, processamento e armazenagem para ELI-Spot

A partir da consulta do dia 42 o esquema de acompanhamento dos voluntários com infecção pelo HIV será diferente daquele a ser seguido para os voluntários HIV negativos, a fim de permitir avaliações a cada 8 semanas tanto após a primeira dose quanto após o esquema vacinal completo contra influenza AH1N1 em ambos os grupos estudados.

6.2.9 Consultas de oito semanas (2 meses) após esquema vacinal completo ou após primeira dose contra H1N1: dia 57 (todos) e dia 77 (apenas HIV positivos)
Essa consulta deve ser realizada 8 semanas após a aplicação do esquema vacinal completo, idealmente no mesmo dia da semana (por exemplo, na terceira segunda-feira seguinte à primeira dose para os voluntários inicialmente vacinados numa segunda-feira), sendo permitida uma janela de mais ou menos 7 dias. Os seguintes procedimentos devem ser realizados nessa consulta:

- Atualização de história médica
- Exame físico dirigido e sinais vitais
- Captura e registro de eventos adversos
- Captura e registro de medicações concomitantes
- CD4 e CV (para voluntários infectados pelo HIV, apenas na consulta de dia 57)
- Coleta de sangue, processamento e armazenagem para sorologia (H1N1)

6.2.10 .Consultas de dezesseis semanas (4 meses) após esquema vacinal completo ou após primeira dose contra H1N1: dia 113 (todos) e dia 133 (apenas HIV positivos).

Essa consulta deve ser realizada 16 semanas após a aplicação do esquema vacinal completo, idealmente no mesmo dia da semana (por exemplo, na terceira segunda-feira seguinte à primeira dose para os voluntários inicialmente vacinados numa segunda-feira), sendo permitida uma janela de mais ou menos 7 dias. Os seguintes procedimentos devem ser realizados nessa consulta:

- Atualização de história médica
- Exame físico dirigido e sinais vitais
- Captura e registro de eventos adversos
- Captura e registro de medicações concomitantes
- Coleta de sangue, processamento e armazenagem para sorologia (H1N1)

6.2.11 .Consultas de vinte e quatro semanas (6 meses) após esquema vacinal completo ou após primeira dose contra H1N1: dia 169 (todos) e dia 189 (apenas HIV positivos).

Essa consulta deve ser realizada 24 semanas após a aplicação do esquema vacinal completo, idealmente no mesmo dia da semana (por exemplo, na terceira segunda-feira seguinte à primeira dose para os voluntários inicialmente vacinados numa segunda-feira), sendo permitida uma janela de mais ou menos 7 dias. Os seguintes procedimentos devem ser realizados nessa consulta:

- Atualização de história médica
- Exame físico dirigido e sinais vitais
- Captura e registro de eventos adversos
- Captura e registro de medicações concomitantes
- CD4 e CV (para voluntários infectados pelo HIV, apenas na consulta de dia 169)
- Coleta de sangue, processamento e armazenagem para sorologia (H1N1)
- Coleta de sangue, processamento e armazenagem para ELI-Spot

6.2.12 .Consultas de trinta e duas semanas (8 meses) após esquema vacinal completo ou após primeira dose contra H1N1: dia 225 (todos) e dia 245 (apenas HIV positivos).

Essa consulta deve ser realizada 16 semanas após a aplicação do esquema vacinal completo, idealmente no mesmo dia da semana (por exemplo, na terceira segunda-feira seguinte à primeira dose para os voluntários inicialmente vacinados numa segunda-feira), sendo permitida uma janela de mais ou menos

7 dias. Os seguintes procedimentos devem ser realizados nessa consulta:

- Atualização de história médica
- Exame físico dirigido e sinais vitais
- Captura e registro de eventos adversos
- Captura e registro de medicações concomitantes
- Coleta de sangue, processamento e armazenagem para sorologia (H1N1)

6.2.13 .Consultas de quarenta semanas (10 meses) após esquema vacinal completo ou após primeira dose contra H1N1: dia 281 (todos) e dia 301 (apenas HIV positivos).

Essa consulta deve ser realizada 40 semanas após a aplicação do esquema vacinal completo, idealmente no mesmo dia da semana (por exemplo, na terceira segunda-feira seguinte à primeira dose para os voluntários inicialmente vacinados numa segunda-feira), sendo permitida uma janela de mais ou menos 7 dias. Os seguintes procedimentos devem ser realizados nessa consulta:

- Atualização de história médica
- Exame físico dirigido e sinais vitais
- Captura e registro de eventos adversos
- Captura e registro de medicações concomitantes
- Coleta de sangue, processamento e armazenagem para sorologia (H1N1)

6.2.14 .Consultas de quarenta e oito semanas (12 meses) após esquema vacinal completo ou após primeira dose contra H1N1: dia 337 (todos) e dia 357 (apenas HIV positivos).

Essa consulta deve ser realizada 48 semanas após a aplicação do esquema vacinal completo, idealmente no mesmo dia da semana (por exemplo, na terceira segunda-feira seguinte à primeira dose para os voluntários inicialmente vacinados numa segunda-feira), sendo permitida uma janela de mais ou menos 7 dias. Os seguintes procedimentos devem ser realizados nessa consulta:

- Atualização de história médica
- Exame físico dirigido e sinais vitais
- Captura e registro de eventos adversos
- Captura e registro de medicações concomitantes
- CD4 e CV (para voluntários infectados pelo HIV, apenas na consulta do dia 337)
- Coleta de sangue, processamento e armazenagem para sorologia (H1N1)
- Coleta de sangue, processamento e armazenagem para ELI-Spot

6.2.15 Consultas para diagnóstico etiológico de quadros respiratórios intercorrentes

Esta consulta deverá ser realizada a qualquer momento em que os voluntários apresentem quadro respiratório agudo após qualquer dose da vacina. Os indivíduos incluídos no estudo serão orientados a procurar o IPEC o mais breve possível após o início de um quadro respiratório sugestivo de influenza (febre acompanhada de tosse e/ou dor de garganta, com qualquer outro sinal ou

sintoma associado). Nesta consulta serão realizados os seguintes procedimentos:

- Coleta de dados sobre sinais e sintomas do quadro respiratório
- Coleta de aspirado de nasofaringe (ANF) ou swab combinado de orofaringe. Imediatamente após a coleta o material deve ser colocado em tubo próprio, com meio de transporte, fornecido pelo estudo e encaminhado para o laboratório de Vírus Respiratórios e do Sarampo do IOC, em condições que respeitem as normas de biossegurança.

A coleta de material deve ser realizada preferencialmente até sete (7) dias após o início dos sintomas respiratórios.

6.2.16 Consulta de término do estudo

Esta consulta deverá ser realizada simultaneamente à última consulta do voluntário no estudo, a qualquer momento em que esta ocorrer. A consulta tem como objetivo registrar o motivo do término da participação (estudo completo ou causas para interrupção prematura). Nos casos de interrupção prematura da participação no estudo os seguintes procedimentos deverão ser realizados:

- Atualização de história médica
- Exame físico dirigido e sinais vitais
- Captura e registro de eventos adversos
- Captura e registro de medicações concomitantes
- CD4 e CV (para voluntários infectados pelo HIV)
- Coleta de sangue, processamento e armazenagem para sorologia (H1N1)
- Coleta de sangue, processamento e armazenagem para ELI-Spot

6.3 Instruções especiais e definições das avaliações

As avaliações do estudo devem ser realizadas de acordo com as instruções, definições e/ou metodologias descritas a seguir. Qualquer alteração só poderá ser implementada após aprovação de uma emenda ao protocolo pelo Comitê de Ética em Pesquisa do IPEC, exceto no caso de algum risco imediato para os sujeitos da pesquisa..:

6.3.1 História médica

Serão obtidos história médica completa e dados demográficos nas consultas de triagem e de dia zero do estudo, incluindo data de nascimento, etnia, queixas atuais, patologias pregressas, antecedentes familiares, hábitos (tabagismo, etilismo, uso de drogas ilícitas), data de diagnóstico da infecção pelo HIV, histórico de uso de anti-retrovirais, medicação concomitante, resultados de exames subsidiários prévios. Em todas as outras consultas do estudo a história médica será atualizada com dados sobre sintomas, sinais, diagnósticos, achados de exame físico e resultados alterados de exames laboratoriais que apareçam durante o período de participação do voluntário. Todos os achados que representarem piora clinicamente significante na condição de um paciente desde a consulta de dia zero ou um novo achado médico serão registrados como eventos adversos.

6.3.2 Exame físico e sinais vitais

Um exame físico completo, incluindo altura (apenas na consulta de triagem) e peso, será realizado nas consultas de triagem e de dia zero. O exame físico completo inclui avaliação de força muscular e de reflexos em mebros superiores e inferiores. Exame físico completo não inclui avaliação de fundo de olho, exame perineal e ginecológico – estas avaliações serão feitas apenas se houver queixas específicas, a critério dos médicos pesquisadores. Todos os achados que representarem piora clinicamente significante na condição de um paciente desde a consulta de dia zero ou um novo achado médico serão registrados como eventos adversos. Exame físico dirigido às queixas dos voluntários e incluindo peso será realizado em todas as outras consultas do estudo. Medidas de sinais vitais, incluindo freqüência cardíaca, freqüência respiratória, pressão arterial e temperatura axilar, serão feitas em todas as consultas do estudo.

6.3.3 Randomização

A equipe do estudo receberá duas listas que ordenam aleatoriamente os pacientes infectados pelo HIV em acompanhamento no IPEC (lista de candidatos), que conterão:

Lista 1: Nomes, ordem de seleção e registros dos pacientes que apresentavam CD4 $\leq$ 200 cels/mm^3 na última contagem realizada (CD4 histórico) e de substitutos.

Lista 2: Nomes, ordem de seleção e registros dos pacientes que apresentavam CD4 > 200 cels/mm^3 na última contagem realizada (CD4 histórico) e de substitutos.

Inicialmente, os pacientes que estiverem entre os 180 primeiros de cada lista de candidatos deverão ser contactados e convidados a participar do estudo e passarão pela avaliação da triagem. Os candidatos da lista que não forem participar do estudo por qualquer motivo (impossibilidade de contato, ausência de consentimento ou que não atendam aos critérios de elegibilidade na avaliação até o momento da randomização) deverão ser substituídos pelo primeiro candidato ainda não convidado de acordo com a ordenação na lista de candidatos. Os que apresentarem contagem de CD4 da triagem em faixa diferente da constante da lista, serão alocados no estrato correspondente à sua contagem atual de células CD4, entrando nesse novo estrato com o número de ordem sequencial seguinte ao do último voluntário que entrou nesse estrato.

Ao final, faltando pacientes em qualquer dos grupos, serão convidados tantos pacientes, respeitando-se a ordenação da lista correspondente de pacientes HIV+, quantos forem necessários para atingir o tamanho da amostra (180 em cada estrato de contagem de células CD4). Este procedimento de convite e preenchimento sequencial dos grupos é conhecido como amostragem inversa e está descrito no item 9.4 deste protocolo.

Assim, os 180 primeiros voluntários de cada estrato de contagem de células CD4 (ou de cada lista de candidatos) deverão ser alocados a um dos grupos de esquema vacinal correspondente ao seu estrato de CD4 no momento da randomização. Para tanto, serão preparados dois conjuntos de envelopes numerados de 001 a 180 e em cada um deles constará a indicação do esquema vacinal a ser aplicado ao voluntário. A indicação constante do envelope respeitará a ordenação aleatória estabelecida no processo de randomização.

No dia da randomização deverá ser considerada a contagem de CD4 colhida na triagem do estudo, ou seja os pacientes mudarão de lista de candidatos caso o resultado de CD4 seja diferente do previsto inicialmente. Nesse caso o paciente deverá ser randomizado através da abertura do próximo envelope (numero sequencial) referente a faixa de CD4 real de triagem.

6.3.4 Exames laboratoriais de segurança

Serão obtidas amostras de sangue para os seguintes exames laboratoriais de segurança, hemograma completo, bilirrubinas totais e frações, TGO e TGP. Os exames de segurança serão feitos nas visitas especificadas na seção 6.2 deste protocolo. Os exames serão realizados de acordo com as técnicas padronizadas no laboratório de análises clínicas do IPEC.

6.3.5 Teste de gravidez

O teste de gravidez será feito através da pesquisa de beta-HCG em sangue ou urina, nas consultas de triagem, dia 1 e dia 21 do estudo.

6.3.6 Teste de HIV

Os candidatos a participar do grupo controle (HIV negativos) serão testados na consulta de triagem, através de teste rápido aprovado no Brasil. O teste será realizado após aconselhamento pré-teste e será seguido por aconselhamento pós-teste. Os candidatos que apresentarem resultado positivo serão excluídos do estudo e serão submetidos a exames confirmatórios, sendo acompanhados pela equipe do protocolo até o resultado final dos exames. Os indivíduos que tiverem confirmado o diagnóstico de infecção pelo HIV serão encaminhados para acompanhamento no ambulatório do IPEC ou em outro local de sua preferência.

6.3.7 Exames de acompanhamento da infecção pelo HIV

Serão obtidas amostras de sangue coletado em EDTA (aproximadamente 5ml para cada exame) para a avaliação das contagens de linfócitos T CD4+ e CD8+ (por citometria de fluxo) e carga viral plasmática do HIV (CV) dos participantes com infecção pelo HIV nos momentos especificados na seção 6.2 deste protocolo. Estes exames serão realizados no laboratório de AIDS e Biologia Molecular do IOC.

6.3.8 Avaliação da resposta sorológica à vacina anti-H1N1

Para realização da sorologia serão colhidos 10mL de sangue em tubo soro gel, que serão centrifugados para separação do soro e congelados a – 20oC.
A fim de determinarmos a presença de anticorpos contra o vírus influenza após

a vacinação dos indivíduos, determinaremos as taxas de soroconversão por inibição da hemaglutinação (HI), confirme descrito anteriormente (Szretter et al, 2006). Assim, será coletada uma amostra de soro dos pacientes antes da vacinação e em diferentes momentos pós-vacinação. Determinaremos a proporção de indivíduos que apresentarem soros negativos antes da vacinação e um titulo ≥ 1:40 após a vacinação, ou que eram soropositivos antes da vacinação e tiveram um aumento de 4 vezes em seus títulos de anticorpos. Em casos nos quais exista a possibilidade para presença de reatividade cruzada entre as respostas de anticorpos contra as vacinas de influenza sazonal e pandêmica, realizaremos testes de microneutralização (Miller et al,. 2010).

O sangue para realização da sorologia será coletado nas consultas especificadas na seção 6.2 deste protocolo. Os exames serão realizados ao final do estudo, em bloco.

6.3.9 Avaliação da resposta imune celular à vacina anti-H1N1

A resposta imune celular dos indivíduos infectados pelo HIV-1 e dos voluntários saudáveis submetidos ao presente protocolo vacinal será avaliada através do método de ELISpot. Este método consiste na análise indireta da resposta imune funcional de linfócitos T frente a um antígeno específico, através da identificação e enumeração de células produtoras de citocinas, classicamente de interferon-gama (IFN-γ), onde cada *spot* desenvolvido representa uma única célula responsiva. Para isto, deverão ser coletados cerca de 30ml de sangue venoso, frente ao anticoagulante heparina, para a obtenção das células mononucleares periféricas, através da separação em ficoll, por gradiente de densidade. As células serão, então, contadas e submetidas à criopreservação em soro fetal bovino 10% DMSO, na concentração de $1x10^7$/ml e estocadas em nitrogênio líquido. Em momento oportuno, as células de cada participante, referentes aos dias zero, 21 e 42 serão descongeladas e avaliadas para a produção dos *spots* frente ao antígeno vacinal e frente à proteína p24 do HIV-1, no caso dos indivíduos infectados, ou ao PPD, para os voluntários saudáveis. O ELISpot será realizado em placas de filtração de 96 poços, cobertas com anticorpos monoclonais de captura anti-IFN-γ (Diaclone, França), na concentração de $1x10^5$ células/poço, em duplicadas para cada estímulo e para a ausência de estímulo. As células serão mantidas em estufa por 40 horas e a secreção de IFN-γ detectada através de anticorpo anti- IFN-γ biotinilado (Diaclone, França). Para a revelação dos *spots* será utilizado o sistema estreptavidina-fosfatase alcalina-NBT-BCIP. Respostas serão consideradas positivas quando o número de células formadoras de *spot* (CSF) /10^6 células mononucleares periféricas for igual ou superior a 50, após subtração das culturas sem estímulo.

O sangue para realização desse teste será coletado nas consultas especificadas na seção 6.2 deste protocolo. Os exames serão realizados ao final do estudo, em bloco.

6.3.10 Avaliação de reatogenicidade

A avaliação de reatogenicidade inclui observação e questões sobre a presença de sinais e sintomas no local da aplicação da vacina (endurecimento, nódulos,

dor, vermelhidão e edema) e sistêmicos (febre > 38oC, sonolência, irritabilidade, perda de apetite, calafrios). A reatogenicidade será avaliada durante uma hora imediatamente aós cada dose da vacina, por consulta telefônica 20 a 36 horas após a vacinação e presencialmente sete dias após a vacinação.

6.3.11 Diagnóstico etiológico dos quadros respiratórios agudos

Isolamento viral

As amostras serão tratadas com antibióticos (200 U de penicilina G potássica, 200ug de estreptomicina, 200ug e anfotericina B) por 60 minutos a temperatura de 4ºC para descontaminação bacteriana. Em seguida, serão inoculadas em tubos com cultura de células (HeLa, MDCK). Utilizaremos cerca de 200µl da amostra, numa incubação por 60 minutos a 34ºC. Após este período, o meio de manutenção será acrescentado (~1mL/tubo): DMEM com 1% de soro fetal bovino para células HeLae MEM com 4ug/mL de Tripsina e 0,5% de albumina para células MDCK. Diariamente, as células serão observadas ao microscópio para a detecção de efeitos citopáticos e, tão logo este seja observado ou, após 7 dias a 35 ºC será feita a coleta do sobrenadante das culturas. Este será titulado por HA no caso de influenza ou PCR em tempo real

Extração de ácidos nucléicos

O RNA/DNA total será extraído do sobrenadante do cultivo celular ou diretamente das amostras clinicas, usando o kit QIAmp Viral RNA mini kit, conforme protocolo fornecido pelo fabricante (QIAgen, Hilden). Ao final do processo, o RNA será eluído em Tampão TE (pH 8,0) e estocado a -70°C, até o momento do uso.

Detecção molecular de vírus respiratórios

A detecção dos vírus VRS, adenovirus, Influenza, Parainfluenza, metapneumovírus humano, coronavirus (OC43, 229E, NL63 e HKU1) e bocavirus será realizada por reação multiplex de PCR em tempo real (rRT-PCR), utilizando o kit comercial Fast-track FTD Respiratory Pathogens®, de acordo com as instruções do fabricante (Fast-track Diagnostics). Suscintamente, o ácido nucléico extraído será adicionado à uma mistura contendo os iniciadores e sondas específicas para cada patógeno, as enzimas para transcrição reversa e amplicação das sequências-alvo e tampão. O gene RNase P será utilizado como controle interno. Os ciclos da RT-PCR em tempo real são constituídos por 50°C por 30 min, 95°C 2min, seguidos de 45 ciclos de 92°C por 15 seg e 55°C por 35 seg.

Sequenciamento automático de nucleotídeos

As amostras positivas para Influenza, na etapa anterior, serão submetidas a RT-PCR, utilizando a enzima Superscript III com Taq platinum e iniciadores específicos para amplificar os segmentos genômicos 4 (HA) e 6 (NA) dos vírus influenza A e B Após purificação e quantificação dos produtos de PCR, a reação de sequenciamento será realizada utilizando o kit “Big Dye Terminator Cycle Sequencing Ready Reaction” (Applied Biosystems). Os produtos serão seqüenciados em ambas as direções e analisados no sequenciador automático ABI PRISM^{TM} 3130XLGenetic Analyzer (Applied Biosystems).

Análise filogenética

As seqüências serão alinhadas utilizado o Programa Clustal X e, para as análises filogenéticas, utilizaremos o programa MEGA 4.1 (Tamura e cols., 2007). As árvores filogenéticas serão reconstruídas usando o método de junção de vizinhos ("Neighbour-Joining")(Saitou e Nei, 1987) e as distâncias evolucionárias serão inferidas pelo método de Kimura 2-parâmetros (Kimura, 1980) - ou outro método que melhor se adeqüe aos dados -, incluindo análise de reamostragem ("bootstrap", mínimo de 1000 replicatas).

Ensaio de atividade enzimática da NA

Os vírus influenza A(H1N1)pdm isolados a partir da cultura celular e obtidos de amostras pré e pós-tratamento com fosfato de oseltamivir serão selecionados para o a realização do ensaio de inibição de NA. O ensaio deverá ser realizado segundo o protocolo desenvolvido por Potier e cols. (1979), com modificações para a detecção pelo método fluorométrico. Este ensaio avalia a 4-metilumbeliferona liberada do substrato fluorogenico, o ácido 2-(4-metilumbeliferil) a d-N-acetilneuraminico (MUNANA), pela atividade enzimática da enzima NAdo vírus influenza. Os vírus obtidos de sobrenadantes de culturas celulares de MDCK ou ovos embrionados serão centrifugados a 800 x g por 10 minutos. A atividade de NA de cada vírus será normalizada antes do teste de inibição. A titulação da atividade de NA, bem como da atividade antiviral do carboxioseltamivir poderá ser realizada também através do kit comercial NA-Star segundo instruções do fabricante (Applied Biosystems).

6.4 <u>Estudo de custo-efetividade</u>

A análise de custo-efetividade a ser conduzida tem como objetivo avaliar comparativamente os benefícios e os custos de diferentes estratégias de vacinação. As estratégias de vacinação sendo comparadas são a vacinação dos pacientes infectados com o HIV com diferentes dosagens. Os benefícios serão medidos em número de casos prevenidos e anos de vida ganhos. Os custos a serem considerados incluem os custos médicos diretos derivados da vacinação assim como os custos médicos não gastos devido aos casos prevenidos. O resultado da análise de custo-efetividade será expresso em termos do custo incremental por unidade de benefício ganho.

Os custos e os benefícios determinados pela vacinação são influenciados por diversos fatores que incluem: a efetividade da vacina, o número de casos esperados, o uso de recursos médicos, os custos da vacinação e do cuidado médico. No caso de uma epidemia por H1N1, que supostamente afetará em determinado momento do tempo, assume-se razoável a análise de custo-efetividade para aquela epidemia. Assim, o horizonte analítico, ou seja, o período de tempo para o qual os benefícios e custos serão computados, assumido é de uma epidemia. A perspectiva adotada será a do Serviço Único de Saúde (SUS). O software TreeAge Pro Suite será utilizado para conduzir a análise.

7.0 QUESTÕES DE GERENCIAMENTO CLÍNICO

7.1 Conduta nas reações no local de aplicação e nas reações alérgicas

Os tipos, intensidade e freqüência das reações adversas locais e de hipersensibilidade esperadas estão listados no anexo III deste protocolo. Não há recomendações específicas para o tratamento das reações adversas locais ou sistêmicas ao produto. O manejo dessas reações deve ser feito a critério ds equipe médica do estudo, com o uso de medicações sintomáticas e procedimentos paliativos e/ou de suporte. A vacinação será feita no IPEC, onde estão disponíveis equipamentos, medicamentos e profissionais qualificados para o pronto-atendimento a reações anafiláticas.

7.2 Outros eventos adversos

Ao longo do estudo (que começa quando o TCLE é assinado), os pesquisadores monitorarão cada paciente para o desenvolvimento de qualquer evidência clínica e/ou laboratorial para identificação de um Evento Adverso (AE). Um evento adverso é definido como qualquer ocorrência médica indesejável em um paciente participante da pesquisa e inclui eventos/ experiências mesmo sem relação causal com as vacinas do estudo. Antes da administração das vacinas de estudo, apenas os eventos adversos graves e os eventos adversos que o pesquisador considerar como estando relacionados ao desenho do estudo e/ou procedimentos serão capturados na ficha clínica de estudo.

Um evento adverso pode ser um sintoma, sinal, ou achado de laboratório anormal. Qualquer piora de uma condição pré-existente ou doença intercorrente será relatada como um evento adverso. Uma anormalidade de laboratório será informada como um evento adverso quando for necessária uma conduta (p.ex., interrupção ou descontinuação da droga do estudo, ou necessidade de tratamento específico). A natureza do sinal ou sintoma, sua data e horário de aparecimento, duração e severidade, terapia empregada (se alguma) e a opinião do pesquisador dobre a relação de causalidade com a droga do estudo, com uma etiologia alternativa, se apropriado, será documentada.

Todos os eventos adversos serão acompanhados até a resolução clínica satisfatória.

Os eventos adversos esperados estão descritos no anexo III deste protocolo de pesquisa, incluindo efeitos indesejáveis sistêmicos e no local da aplicação da vacina. Dentre estes, o mais preocupante pela gravidade, apesar de raro, é a Síndrome de Guillain-Barré. A Síndrome de Guillain-Barré não tem sido considerada uma complicação típica da vacinação contra a influenza sazonal, no entanto um maior número de casos foi relatado em associação com a vacina contra a nova cepa de influenza suína de 1976 que levou à imunização em massa em preparação para uma possível pandemia. A vigilância nacional descobriu um total de 1.098 pacientes com início de síndrome de Guillain-Barré de 1 de outubro de 1976 a 31 de janeiro de 1977. No total, 532 pacientes

tinham recebido recentemente vacinação contra influenza A/New Jersey antes do início da síndrome de Guillain-Barré. O risco estimado de síndrome de Guillain-Barré relacionada com a vacina na população adulta foi ligeiramente inferior a um caso por 100.000 vacinações. Esta experiência vai ditar algumas características da avaliação de segurança prevista para este protocolo.

O pesquisador classificará a intensidade do evento adverso de acordo com a definição da tabela de "Graus de Toxicidade Clínica" (anexo II). Esta tabela será usada na gradação dos eventos adversos para o controle da toxicidade. Os eventos não previstos na tabela terão sua intensidade classificada de acordo com os seguintes parâmetros:

Leve:	O evento adverso é passageiro e facilmente tolerado pelo paciente.
Moderado:	O evento adverso causa desconforto ao paciente e interrompe suas atividades normais.
Severo:	O evento adverso causa interferência considerável com as atividades normais do paciente, podendo ser incapacitante ou representar um risco de vida.

Os pesquisadores usarão as definições a seguir para avaliar a relação do evento adverso com as vacinas do estudo:

Provável:	O evento adverso tem uma relação temporal forte com a vacina do estudo ou recorre após a segunda dose, e uma outra etiologia é improvável ou significativamente menos provável.
Possível:	O evento adverso tem uma relação temporal forte com a vacina do estudo, e uma etiologia alternativa é igualmente ou menos provável comparado à relação potencial com a droga do estudo. A etiologia alternativa deve ser fornecida pelo pesquisador.
Provavelmente Não:	O evento adverso tem pequena ou nenhuma relação temporal com a vacina do estudo e/ou existe uma etiologia alternativa mais provável. A etiologia alternativa deve ser fornecida pelo pesquisador.
Não Relacionado:	O evento adverso é decorrente de uma doença de base ou simultânea ou efeito de outra droga e não está relacionado à vacina do estudo. A etiologia alternativa deve ser fornecida pelo pesquisador.

7.2.1 Eventos Adversos Graves

Eventos adversos graves serão relatados ao grupo coordenador médico do estudo dentro de 24 horas do conhecimento da ocorrência pelo pesquisador e ao Comitê de Ética em Pesquisa o mais breve possível. Os eventos devem ser relatados através do preenchimento de um formulário específico, enviado por e-mail para o grupo coordenador médico.

Um Evento Adverso Grave (EAG) é uma experiência adversa à droga que resulta em alguma das seguintes evoluções:

- **Morte.**
- **Situação com risco de vida -** O paciente está sob risco de vida no momento do evento/experiência adversa. Não se refere a um risco hipotético de morte se o EA fosse mais grave ou progredisse.
- **Hospitalização do paciente ou prolongamento de uma hospitalização existente.**
- **Incapacidade/ invalidez persistente ou significativa -** Todo EA que tiver uma evolução que esteja associada com uma ruptura substancial da capacidade de conduzir as funções normais da vida, incluindo a capacidade para trabalhar. Não estão incluídas interrupções transitórias das atividades diárias.
- **Anomalia congênita/ defeitos ao nascimento -** Qualquer anormalidade estrutural em um filho do paciente que ocorra depois da exposição intra-uterina ao tratamento.
- **Eventos/experiências médicas importantes** que possam não resultar em morte, ser um risco de vida, ou precisar de hospitalização podem ser considerados sérios quando, baseado em julgamento médico apropriado, **eles possam comprometer o paciente e possam precisar de intervenção médica ou cirúrgica para prevenir uma das evoluções listadas acima,** i.é., morte, evento/experiência adversa com risco de vida, hospitalização do paciente ou prolongamento de uma hospitalização já existente, incapacidade/ invalidez persistente ou significativa, ou uma anomalia congênita/defeito ao nascimento. Exemplos de tais eventos/experiências médicas incluem broncoespasmo alérgico que precisa de tratamento intensivo em pronto socorro ou em casa, discrasias sangüíneas ou convulsões que não resultam em hospitalização.

7.3 Gestação

Não há dados disponíveis sobre o uso de Pandemrix® durante a gravidez. Estudos com vacinas compostas por vírus inativado, sem adjuvantes, contra influenza sazonal não demonstraram relação do produto com malformações, toxicidade fetal ou neonatal. Estudos pré-clínicos não demonstraram toxicidade reprodutiva de Pandemrix®.

Mulheres grávidas no momento da triagem e na consulta de dia zero não serão incluídas neste estudo. Mulheres com teste positivo para gravidez no dia da aplicação da segunda dose de Pandemrix® e ou da vacina contra influenza sazonal não receberão o produto, porém permanecerão no estudo para análise de segurança e de imunogenicidade, cumprindo os demais procedimentos do estudo.

8.0 CRITÉRIOS DE RETIRADA DO ESTUDO

8.1 Interrupção do esquema vacinal com permanência no estudo

Voluntários com eventos adversos atribuídos à vacina de grau maior ou igual a 3, ou que desenvolvam intercorrências clínicas relevantes, não receberão a segunda dose da vacina, porém permanecerão no estudo para análise de segurança e de imunogenicidade, cumprindo os demais procedimentos previstos neste protocolo.

Os voluntários que se apresentarem para a segunda dose da vacina após o prazo determinado não receberão essa dose, mas permanecerão no estudo para análise de segurança e de imunogenicidade, cumprindo os demais procedimentos previstos neste protocolo.

8.2 Saída prematura do estudo

Qualquer paciente participante desse estudo poderá retirar seu consentimento, a qualquer momento e por qualquer motivo, sem que isso implique em prejuízo para seu tratamento nas instituições envolvidas. Os pesquisadores poderão retirar qualquer indivíduo do estudo, por motivos que julgaram pertinentes (toxicidade, falta de adesão, etc).

Esse estudo pode ser prematuramente interrompido pelos pesquisadores, pelo CEP do IPEC ou pelos órgãos regulatórios brasileiros, caso seja demonstrado risco para a segurança dos sujeitos da pesquisa.

9.0 CONSIDERAÇÕES ESTATÍSTICAS

9.1 Questões gerais do desenho do estudo

Este é um ensaio clínico randomizado, aberto, de não-inferioridade, para avaliar a imunogenicidade, a reatogenicidade, a segurança e a tolerabilidade de dois esquemas vacinais (descritos no item 3 deste protocolo) contra o H1N1 em pacientes adultos infectados pelo HIV, em comparação com o esquema vacinal proposto para indivíduos HIV negativos.

Além dos dois esquemas vacinais, será controlada a contagem de células CD4 dos pacientes infectados pelo HIV formando dois estratos de contagem de CD4 (≤ 200 cels/mm3 ou > 200 cels/mm3).

Dessa forma, serão quatro grupos ou estratos definidos pela combinação dos estratos de contagem de células CD4 e pelo esquema vacinal, além do grupo de indivíduos HIV negativos.

A população de pesquisa será constituída pelo conjunto de pacientes em tratamento no Instituto de Pesquisa Clínica Evandro Chagas (IPEC/Fiocruz), que estejam sem receber tratamento anti-retroviral ou em tratamento estável há

no mínimo 8 semanas, sem planos de mudança nos próximos 6 meses, com indicação de receber vacina contra influenza A H1N1. O grupo controle será formado por indivíduos HIV negativos, confirmado por teste no momento da triagem, com indicação de receber vacina contra influenza A H1N1.

Os critérios de inclusão e exclusão foram descritos no item 4 deste protocolo.

9.2 Desfechos

O estudo foi desenhado para medir as proporções de soroconversão e soroproteção e o fator de proteção da vacina contra influenza A H1N1 em cada um dos quatro subgrupos de portadores do HIV e no grupo de controle, a fim de testar a não-inferioridade de cada estrato em relação ao grupo de controle.

As medições seguirão o calendário descrito no item 6.2 e as estimativas e testes serão feitos tão logo estejam disponíveis os resultados das sorologias.

9.3 Estratificação

Os portadores de HIV serão estratificados de acordo com o resultado do exame de contagem de células CD4 feito no dia da triagem em dois estratos: (1) CD4 menor ou igual a 200 cels/mm^3;e (2) CD4 maior que > 200 cels/mm3.

Em seguida, será feita a randomização que indicará para qual esquema vacinal (2+2 doses ou 1+1 dose) cada indivíduo será alocado.

9.4 Tamanho da amostra, seleção e recrutamento

O tamanho da amostra foi calculado para um estudo de não-inferioridade levando em consideração os seguintes parâmetros: proporção de soroconversão de 0,95, diferença aceitável para não inferioridade de 10%, nível de significância de 0,05 e potência do teste de equivalência de 90%.

Esses resultados conduziram a um tamanho de amostra de 82 pessoas em cada grupo, que foi ampliado para 90 a fim de compensar as perdas previstas ao longo do ensaio clínico.

A seleção dos possíveis participantes será feita a partir da lista de portadores de HIV em tratamento no IPEC. Os indivíduos constantes da lista serão estratificados de acordo com o último resultado da contagem de células CD4 registrada no banco de dados do laboratório de Pesquisa Clínica em DST e AIDS nos dois estratos acima definidos. Em cada estrato os pacientes serão ordenados por sexo e idade e um processo de seleção sistemática de 180 indivíduos (e 180 substitutos) será realizado.

A escolha desse método de seleção aleatória deve-se ao fato de que a combinação da seleção sistemática com a ordenação prévia do cadastro de seleção corresponde a uma estratificação implícita da população por sexo e idade, que assegurará que os grupos de participantes tenham aproximadamente a mesma distribuição por sexo e.idade da população de pesquisa.

Posteriormente, os 180 candidatos serão ordenados aleatoriamente, para estabelecer a ordenação da lista de candidatos de cada estrato de contagem de células CD4. O mesmo será feito com a lista de substitutos de cada estrato, que será copiada em continuação à lista com os primeiros 180 candidatos selecionados.

No momento da triagem será feito o exame de CD4. Com seu resultado, a classificação nos estratos de contagem de células CD4 será confirmado. Caso o participante tenha contagem incompatível com o estrato em que foi selecionado, ele será, então, realocado ao estrato correspondente ao resultado de seu exame.

Este procedimento de seleção visa a considerar as ocorrências decorrentes dos critérios de elegibilidade e exclusão, que conduzirão a que alguns candidatos selecionados sejam não-elegíveis para o estudo, assegurando que o tamanho da amostra de cada estrato de contagem de células CD4 seja de 180 indivíduos.

Para tanto, será necessário aplicar um procedimento seqüencial de recrutamento, principalmente quando os grupos estiverem próximos de atingir o tamanho de sua amostra. Este procedimento sequencial é conhecido como amostragem inversa (Haldane, 1945; Vasconcellos, Silva & Szwarcwald, 2005). Na realidade, esse é um procedimento seqüencial derivado de uma ordenação aleatória prévia, que termina quando o número de participantes do grupo (90, no caso) for atingido.

Para o grupo de controle, serão selecionados os primeiros 90 candidatos que aceitarem participar do estudo e cumprirem os demais critérios de elegibilidade.

A grande vantagem desse processo de seleção de candidatos reside no fato de que serão conhecidas as probabilidade de inclusão no estudo dos participantes e, portanto, podem ser estimadas prevalências para combinação de estratos, que podem ser testadas com as do grupo de controle.

9.5 Monitoramento

Os pesquisadores responsáveis, a equipe de gerenciamento de dados e os estatísticos do estudo avaliarão semanalmente as listas de randomização e a lista de portadores do HIV previamente selecionados para participação no estudo, de acordo com os estratos de CD4, a fim de monitorar a velocidade de inclusão e de preenchimento dos subgrupos do estudo. Serão analisados também o número de participantes que consentiu em participar do estudo e o número de sujeitos que mudaram de estrato devido à contagem de CD4 no momento da triagem para o estudo, além do número de exclusões por critérios de elegibilidade. Essa análise freqüente permitirá eventuais correções nas listas de seleção, mantendo a aleatoriedade do procedimento de seleção.

9.6 Análises

Será feita uma análise demográfica de cada grupo e dos quatro grupos de portadores do HIV, sendo calculadas, por sexo, as médias e desvios padrão de idade, que serão comparados aos mesmos parâmetros do conjunto de pacientes HIV positivos em tratamento no IPEC. Os grupos randomizados e o grupo de voluntários HIV-negativos terão suas características pré-vacinais (sócio-demográficas, clínicas e laboratoriais) comparadas, para demonstrar o sucesso da randomização e a comparabilidade do controle não randomizado. O conjunto de pacientes randomizados poderá ser comparado em algumas características selecionadas com o "universo" dos pacientes do IPEC, como um acessório para analisar a validade externa dos resultados.

A não-inferioridade da vacina contra gripe A H1N1 em portadores do HIV, em relação ao grupo de controle, será avaliada a partir da proporção de soroproteção obtida com as sorologias nos dias indicados no item 6.2 deste protocolo.

Os intervalos de 95% de confiança das diferenças entre as proporções de soroproteção entre os grupos de portadores do HIV e o grupo de controle serão construídos e a não-inferioridade será definida como limite inferior do intervalo de confiança de 95% para a diferença na proporção de soroproteção entre os grupos de HIV positivos e o de controle de -10% (por exemplo, -8%, -5% etc.).

De forma análoga, será testada a não-inferioridade entre os dois esquemas vacinais propostos para cada estrato de contagem de células CD4.

Como os estratos podem ser grupados e a proporção de soroproteção estimada para esses grupamentos, testes de não-inferioridade podem ser conduzidos para estratos agrupados e para o conjunto de participantes HIV positivos.

Similarmente, as razões das médias geométricas dos títulos de anticorpos nos grupos HIV positivo e no grupo de referência e seus respectivos intervalos de 95% de confiança serão estimados. Não-inferioridade será definida como limite inferior da razão de títulos médios geométricos de anticorpos contra gripe A H1N1 ao final do esquema completo em cada par de grupos em comparação, superior a 0,5 (por exemplo, 0,6, 0,7 etc.).

A análise de segurança será realizada através da determinação, por dose, da proporção de sujeitos com um relato de qualquer sintoma durante a avaliação de segurança (item 6.2). Para cada grupo e dose, a incidência de cada sintoma observado será apurada. Serão tabuladas as proporções de eventos adversos por dose e por grupo, que serão comparadas através de teste de qui-quadrado com nível de significância de 0,01.

A duração da resposta humoral à vacina será avaliada através da comparação da freqüência de sujeitos com títulos protetores de anticorpos em cada momento em que a sorologia for realizada, entre 2 e 12 meses após a vacinação

10.0 PLANO FARMACOLÓGICO

10.1 Negociação com o Ministério da Saúde (vacinas do mesmo fabricante) – Os pesquisadores acordaram com o Ministério da Saúde que o IPEC receberá doses suficientes da vacina para serem usadas durante todo o estudo, de um mesmo fabricante.

10.2 Transporte do produto vacinal
A conservação de uma vacina é feita através do sistema de cadeia de frio que inclui o armazenamento, o transporte, a manipulação das vacinas e as condições de refrigeração, desde o laboratório produtor até o momento em que a vacina é aplicada. As vacinas do estudo devem ser transportadas na faixa de temperatura de 2-8ºC. Deve haver um controle contínuo da temperatura durante o transporte através de um data logger. O farmacêutico ao receber as vacinas deve se certificar de que não houve oscilação de temperatura fora da faixa permitida. Em caso de variação de temperatura fora da faixa de 2-8ºC, o farmacêutico deve manter as vacinas em quarentena e entrar em contato imediatamente com o fornecedor.

10.3 Controle de estoque
O farmacêutico deve manter o inventário dos produtos atualizado, utilizando um formulário de contabilidade para registrar cada dispensação do produto em investigação ou a cada novo envio do fornecedor. O farmacêutico deve realizar um inventário físico pelo menos uma vez ao mês, a fim de verificar se a quantidade real corresponde a quantidade documentada.

10.4 Dispensação
A dispensação dos produtos do estudo deve ser feita diretamente ao profissional de saúde pertencente a equipe do estudo que irá realizar a administração no paciente. A dispensação só poderá ser feita mediante prescrição médica, assinada por um prescritor autorizado pelo investigador principal. O farmacêutico deve assegurar que o termo de consentimento livre e esclarecido foi assinado antes de dispensar os produtos do estudo. Todo procedimento de dispensação deve ser documentado no prontuário do paciente.

11.0 COLETA DE DADOS E CONTROLE E NOTIFICAÇÃO DE EVENTOS ADVERSOS

11.1 Registros a serem mantidos

Para cada paciente que assinar o TCLE e se submeter aos procedimentos de seleção para este estudo, será completada uma ficha clínica de estudo, especificamente desenvolvida para esse estudo. Isto se aplica também aos pacientes que se submeterem aos procedimentos de seleção mas não foram admitidos e aos pacientes que forem descontinuados antes da conclusão do

estudo. A razão pela qual um paciente não foi admitido ou a razão para descontinuação será descrita no documento fonte e na FCE.

Todas as fichas clínicas de estudo serão completadas com uma caneta esferográfica de cor escura, em letra legível. As correções serão feitas traçando uma única linha na entrada incorreta e escrevendo a entrada correta. Todas as correções serão datadas e rubricadas.

Qualquer referência a pacientes será feita usando-se suas iniciais e números específicos do protocolo, nunca pelos nomes.

O pesquisador responsável pela pesquisa ou um profissional da equipe do estudo revisará as fichas clínicas de estudo para verificar a precisão dos dados coletados.

Será montado um banco de dados específico para o estudo e os dados constantes das FCEs serão transferidos para este banco utilizando-se o software Teleform®. A entrada dos dados no banco será feita na Unidade Central de Dados do Laboratório de Pesquisa Clínica em DST e AIDS do IPEC – FIOCRUZ. Um profissional da equipe da central de dados fará controle de qualidade dos dados inseridos no banco, incluindo verificações internas de inconsistências e geração de questionamentos para os pesquisadores para eventuais correções que sejam necessárias.

11.2 Papel do gerenciamento de dados

A Unidade Central de Dados do Laboratório de Pesquisa Clínica em DST e AIDS fornecerá à equipe de pesquisadores as instruções referentes ao registro dos dados do estudo nas FCE. Cada pesquisador é responsável pela inserção dos dados em tempo hábil para as análises previstas.

É de responsabilidade da Unidade Central de Dados assegurar a qualidade dos dados de informática desse estudo. Esta responsabilidade se estende desde o desenvolvimento do protocolo até a criação dos bancos de dados finais do estudo.

11.3 Monitoramento do centro clínico e disponibilidade dos registros

O estudo tem um grupo médico coordenador responsável por:

- responder dúvidas dos demais pesquisadores sobre os procedimentos do estudo
- organizar e participar das análises preliminares dos dados obtidos, redigindo eventuais emendas ao protocolo original

- avaliar constantemente a condução do estudo em seus aspectos éticos e científicos, promovendo alterações nos procedimentos sempre que necessário

- avaliar eventos adversos graves imediatamente após sua ocorrência

- notificar os demais pesquisadores sobre o andamento do estudo

Durante o desenvolvimento do estudo não está previsto monitoramento externo de qualidade.

As equipes de controle de qualidade e da central de dados do Laboratório de Pesquisa Clínica em DST e AIDS avaliarão os seguintes parâmetros de qualidade dos dados:**:**

- erros de transcrição das informações nas Fichas Clínicas de Estudo (FCE) - verificação de 20% dos prontuários e FCE;
- se todos as FCEs foram preenchidss por consulta e por voluntário;
- se as FCES referentes as consultas foram enviados a Central de Dados
- se todos os questionamentos da central de dados foram respondidos adequadamente

Na etapa de Controle de Segurança são avaliados:

- se os procedimentos foram realizados e registrados adequadamente - verificação de 20% dos prontuários e FCE
- se os TCLE foram adequadamente assinados – revisão de 100% dos termos;
- se os eventos adversos graves foram relatados no prazo previsto e de forma completa e acurada – revisão de 100% dos relatos;
- se os documentos regulatórios foram gerados e arquivados de forma correta – revisão de 100% dos arquivos do pesquisador

Todos os registros do estudo estarão disponíveis para a equipe de pesquisadores, o CEP do IPEC, a CONEP e a ANVISA.

11.4 <u>Eventos adversos de notificação imediata para o grupo coordenador médico do estud</u>o

Todos os eventos adversos graves deverão ser comunicados em até 24 horas após o conhecimento para o grupo coordenador médico do estudo, através de formulário específico de notificação enviado por e-mail ao grupo. Também deverão ser notificados no mesmo formulário os eventos adversos locais e sistêmicos da vacina de grau maior ou igual a 3.

12.0 PARTICIPANTES HUMANOS

12.1 Revisão do Comitê de Ética em Pesquisa (CEP) e termo de consentimento livre e esclarecido

Este protocolo só será iniciado após avaliação e aprovação pelo CEP do IPEC.

Será considerada responsabilidade dos pesquisadores assegurar que a cada paciente foi dada uma explicação adequada dos objetivos, métodos, benefícios antecipados e riscos potenciais deste estudo e que cada participante voluntariamente assine e date o TCLE aprovado pelo CEP antes da participação no estudo (anexo III). O pesquisador também deverá explicar que os pacientes têm o direito de se recusar a participar no estudo ou de se retirar a qualquer momento por qualquer razão.

Será documentado no registro médico da paciente que o consentimento informado foi obtido antes da realização de qualquer procedimento relacionado ao estudo e que uma via original do TCLE foi mantida com os registros do estudo. Uma via do TCLE assinado e datado será oferecida aos pacientes.

12.2 Confidencialidade da participante

Os pesquisadores manterão um diário de seleção/ admissão e informação de identificação completa sobre cada paciente a ser usado com a finalidade de seguimento a longo prazo, se necessário. Este documento será mantido pelos pesquisadores em sigilo estrito.

As fichas clínicas de estudo serão identificadas apenas com o número do paciente no estudo. Qualquer divulgação dos resultados desta pesquisa não conterá nomes, fotos ou outras formas de identificação dos sujeitos da pesquisa.

12.3 Término do estudo

O estudo pode ser terminado a qualquer momento pelo CEP, a CONEP, a ANVISA ou outros órgãos do governo como parte de seus deveres de assegurar a proteção dos participantes de pesquisas.

13.0 PUBLICAÇÃO DAS DESCOBERTAS DA PESQUISA

Os dados obtidos com esse estudo são de propriedade dos pesquisadores responsáveis. A publicação dos resultados das análises será feita, independente de serem favoráveis ou não, após acordo entre os pesquisadores sobre autoria. Esse estudo não envolve cooperação estrangeira.

14.0 CONTENÇÃO DE RISCO BIOLÓGICO

Todos os procedimentos de manipulação das amostras biológicas dos indivíduos infectados pelo HIV-1 e pelos voluntários saudáveis seguirão as

recomendações de boas práticas estabelecidas pela CIBio – IOC/FIOCRUZ. As amostras biológicas obtidas para os exames de acompanhamento e de avaliação imunológica deste protocolo, tanto dos indivíduos infectados pelo HIV-1 como dos voluntários saudáveis, serão avaliados nos laboratórios de segurança biológica de nível 3 (NB3) do IOC ou no laboratório de segurança biológica de nível 2 (NB2) do Laboratório de AIDS e Imunologia Molécula – IOC. Todo o material consumível utilizado em todas as etapas deste protocolo será devidamente autoclavado e descartado segundo as normas de biossegurança regidas na Fiocruz.

Nas visitas interinas do estudo para avaliação clínica e coleta de secreção respiratória para identificação da etiologia do quadro respiratório serão seguidos os procedimentos de segurança recomendados pelo Ministério da Saúde do Brasil e padronizados no IPEC.

15.0 REFERÊNCIAS

AMENDOLA A, Boschini A, Colzani D, et al. InflUenza vaccination of HIV-1-positive and HIV-1-negative former intravenous drug users. *J Med Virol* 2001; 65: 644–48.

ANEMA A, Mills E, Montaner J, Brownstein JS, Cooper C. Efficacy of influenza vaccination in HIV-positive patients: a systematic review and meta-analysis. *HIV Med* 2008; 9: 57–61.

ATASHILI J, Kalilani L, Adimora AA. Efficacy and clinical effectiveness of influenza vaccines in HIV-infected individuals: a meta-analysis. BMC Infect Dis 2006; 6:138.

ATMAR RL, Keitel WA, In Compans R.W., et.al. Adjuvants for pandemic influenza vaccines. Vaccines for Pandemic Influenza. Current Topics in Microbiology. Springer Verlag: Berlin, 2009, in press, 2009.

ATMAR RL, Keitel WA, Patel SM et al. Safety and immunogenicity of nonadjuvanted and MF59-adjuvanted influenza A/H9N2 vaccine preparations. Clin Infect Dis 2006; 43(9):1135-1142

BRUGUERA M, Cremades M, Salinas R, Costa J, Grau M. Impaired response to recombinant hepatitis B vaccine in HIV-infected persons. J Clin Gastroenterol 1992;14(1):27-30

BRUGUERA M, Rodicio JL, Alcazar JM, Oliver A, Del Rio G, Esteban-Mur R. Effects of different dose levels and vaccination schedules on immune response to a recombinant DNA hepatitis B vaccine in haemodialysis patients. Vaccine 1990;8 Suppl:S47-9; discussion S60-2

BRYDAK LB, Hryniewicz HJ, Machala M, Horban A. Humoral response to influenza vaccination in HIV-infected patients. *Clin Drug Invest* 1999; 17: 441–49.

CENTERS for Disease Control and Prevention (CDC). Serum cross-reactive antibody response to a novel influenza A (H1N1) virus after vaccination with seasonal influenza vaccine. MMWR Morb Mortal Wkly Rep 2009; 58(19):521-524

CHADWICK EG, Chang G, Decker MD, Yogev R, Dimichele D, Edwards KM. Serologic response to standard inactivated influenza vaccine in human immunodeficiency virus-infected children. Pediatr Infect Dis J 1994; 13(3):206-211

COUCH RB, Kasel JA. Immunity to influenza in man. Annu Rev Microbiol 1983; 37:529-549.

DAWOOD FS, Jain S, Finelli L et al. Emergence of a novel swine-origin influenza A (H1N1) virus in humans. N Engl J Med 2009; 360(25):2605-2615

DIVISION of AIDS, Pharmaceutical Affairs Branch, Pharmacy Guidelines and Instructions for DAIDS Clinical Trials Networks, July 2008

ENNIS FA, Mayner RE, Barry DW et al. Correlation of laboratory studies with clinical responses to A/New Jersey influenza vaccines. J Infect Dis 1977; 136 Suppl:S397-S406

EVISON J Farese S, Seitz M, Uehlinger D, Furrer H, Mühlemann K. Randomized, double-blind comparative trial of subunit and virosomal influenza vaccines for immunocompromised patients. *Clin Infect Dis* 2009; 48: 1402–12.

FALSEY AR, Treanor JJ, Tornieporth N, Capellan J, Gorse GJ. Randomized, double-blind controlled phase 3 trial comparing the immunogenicity of high-dose and standard-dose influenza vaccine in adults 65 years of age and older. J Infect Dis 2009; 200(2):172-180.17;19;20)

FINE AD, Bridges CB, De Guzman AM et al. Influenza A among patients with human immunodeficiency virus: an outbreak of infection at a residential facility in New York City. Clin Infect Dis 2001; 32(12):1784-1791.

FINE AD, Bridges CB, De Guzman AM, et al. Influenza A among patients with human immunodeficiency virus: an outbreak of infection at a residential facility in New York City. *Clin Infect Di*s 2001; 32: 1784–91.

FLYNN PM, Wilson RB, Kapogiannis BG et al. Improving Seroresponse to Hepatitis B Vaccination in HIV-Infected Adolescents: ATN 024. International AIDS Society, Capetown, SA, July 19-22, 2009 and 1st International Workshop on HIV in Pediatrics, Capetown, SA, July 17-18, 2009

FOWKE KR, D'Amico R, Chernoff DN, et al. Immunologic and virologic evaluation after influenza vaccination of HIV-1-infected patients. *AIDS* 1997; 11: 1013–21.

GALLI G, Hancock K, Hoschler K et al. Fast rise of broadly cross-reactive antibodies after boosting long-lived human memory B cells primed by an MF59 adjuvanted prepandemic vaccine. Proc Natl Acad Sci U S A 2009; 106(19):7962-7967

GATHERER D. The 2009 H1N1 influenza outbreak in its historical context. J Clin Virol 2009; 45(3):174-178

GATHERER D. The 2009 H1N1 influenza outbreak in its historical context. J Clin Virol 2009; 45(3):174-178.

GELINCK LB, van den Bemt BJ, Marijt WA et al. Intradermal influenza vaccination in immunocompromised patients is immunogenic and feasible. Vaccine 2009; 27(18):2469-2474

GROSS PA, Quinnan GV, Jr., Weksler ME, Gaerlan PF, Denning CR. Immunization of elderly people with high doses of influenza vaccine. J Am Geriatr Soc 1988; 36(3):209-212

HACKETT S, Hill L, Patel J et al. Clinical characteristics of paediatric H1N1 admissions in Birmingham, UK. Lancet 2009;(374):605

HALDANE JBS. On a method of estimating frequencies. Biometrika 1945; 33:222-5

HAMMITT LL, Li S, Patterson-Bartlett J, et.al. Kinetics o fviral shedding and immune responses to cold-adapted influenza vaccine. 10th International Symposium on Respiratory Viral Infections, Singapore, Thailand, Feb 28-Mar 2, 2008

HOLLAND D, Booy R, De LF et al. Intradermal influenza vaccine administered using a new microinjection system produces superior immunogenicity in elderly adults: a randomized controlled trial. J Infect Dis 2008; 198(5):650-658

IORIO AM, Alatri A, Francisci D, et al. Immunogenicity of influenza vaccine (1993–94 winter season) in HIV-seropositive and -seronegative ex-intravenous drug users. *Vaccine* 1997; 15: 97–102.

KEET IP, van Doornum G, Safary A, Coutinho RA. Insufficient response to hepatitis B vaccination in HIV-positive homosexual men. AIDS 1992;6(5):509-10

KEITEL WA, Atmar RL, Cate TR et al. Safety of high doses of influenza vaccine and effect on antibody responses in elderly persons. Arch Intern Med 2006; 166(10):1121-1127

KEITEL WA, Atmar RL, Nino D, Cate TR, Couch RB. Increasing doses of an inactivated influenza A/H1N1 vaccine induce increasing levels of cross-reacting antibody to subsequent, antigenically different, variants. J Infect Dis 2008; 198(7):1016-1018

KEITEL WA, Cate TR, Atmar RL et al. Increasing doses of purified influenza virus hemagglutinin and subvirion vaccines enhance antibody responses in the elderly. Clin Diagn Lab Immunol 1996; 3(5):507-510

KEITEL WA, Couch RB, Cate TR et al. High doses of purified influenza A virus hemagglutinin significantly augment serum and nasal secretion antibody responses in healthy young adults. J Clin Microbiol 1994; 32(10):2468-2473

KING JC, Jr., Treanor J, Fast PE et al. Comparison of the safety, vaccine virus shedding, and immunogenicity of influenza virus vaccine, trivalent, types A and B, live cold-adapted, administered to human immunodeficiency virus (HIV)-infected and non-HIV-infected adults. J Infect Dis 2000; 181(2):725-728

KROON FP, Rimmelzwaan GF, Roos MT, et al. Restored humoral immune response to influenza vaccination in HIV-infected adults treated with highly active antiretroviral therapy. *AIDS* 1998; 12: F217–23.

KROON FP, van Dissel JT, de Jong JC, van FR. Antibody response to influenza, tetanus and pneumococcal vaccines in HIV-seropositive individuals in relation to the number of CD4+ lymphocytes. AIDS 1994; 8(4):469-47

LEROUX-ROELS I, Vets E, Freese R et al. Seasonal influenza vaccine delivered by intradermal microinjection: A randomized controlled safety and immunogenicity trial in adults. Vaccine 2008; 26(51):6614-6619

LEVIN MJ, Song LY, Fenton T et al. Shedding of live vaccine virus, comparative safety, and influenza-specific antibody responses after administration of live attenuated and inactivated trivalent influenza vaccines to HIV-infected children. Vaccine 2008; 26(33):4210-4217

MADHI SA, Ramasamy N, Bessellar TG, Saloojee H, Klugman KP. Lower respiratory tract infections associated with influenza A and B viruses in an area with a high prevalence of pediatric human immunodeficiency type 1 infection. Pediatr Infect Dis J 2002; 21(4):291-297.

MATZKIN H, Nili E. Accidental tenfold overdose of influenza vaccine: a clinical and serological study. Isr J Med Sci 1984; 20(5):411-415

MENDOZA-SANCHEZ MC, Ruiz-Contreras J, Vivanco JL et al. Respiratory virus infections in children with cancer or HIV infection. J Pediatr Hematol Oncol 2006; 28(3):154-159

MILLER E, Hoschler K, Hardelid P, Stanford E, Andrews N, Zambon M. Incidence of 2009 pandemic influenza A H1N1 infection in England: a cross-sectional serological study. Lancet. 2010 Jan 20.

MIOTTI PG, Nelson KE, Dallabetta GA, Farzadegan H, Margolick J, Clements ML. The influence of HIV infection on antibody responses to a two-dose regimen of influenza vaccine. *JAMA* 1989; 262: 779–83.

MIOTTI PG, Nelson KE, Dallabetta GA, Farzadegan H, Margolick J, Clements ML. The influence of HIV infection on antibody responses to a two-dose regimen of influenza vaccine. JAMA 1989; 262(6):779-783
NELSON KE, Clements ML, Miotti P, Cohn S, Polk BF. The influence of human immunodeficiency virus (HIV) infection on antibody responses to influenza vaccines. *Ann Intern Med* 1988; 109: 383–88.
OVERTON ET, Sungkanuparph S, Powderly WG, Seyfried W, Groger RK, Aberg JA. Undetectable plasma HIV RNA load predicts success after hepatitis B vaccination in HIV-infected persons. Clin Infect Dis 2005;41(7):1045-8
OVERTON ET, Sungkanuparph S, Powderly WG, Seyfried W, Groger RK, Aberg JA. Undetectable plasma HIV RNA load predicts success after hepatitis B vaccination in HIV-infected persons. Clin Infect Dis 2005;41(7):1045-8.
OXFORD JS. Influenza A pandemics of the 20th century with special reference to 1918: virology, pathology and epidemiology. Rev Med Virol 2000; 10(2):119-133
PALACHE AM, Beyer WE, Sprenger MJ et al. Antibody response after influenza immunization with various vaccine doses: a double-blind, placebo-controlled, multi-centre, dose-response study in elderly nursing-home residents and young volunteers. Vaccine 1993; 11(1):3-9
PATRIARCA PA, Cox NJ. Influenza pandemic preparedness plan for the United States. J Infect Dis 1997; 176 Suppl 1:S4-S7
PEIRIS JS, Poon LL, Guan Y. Emergence of a novel swine-origin influenza A virus (S-OIV) H1N1 virus in humans. J Clin Virol 2009; 45(3):169-1731-4
PEIRIS JS, Poon LL, Guan Y. Emergence of a novel swine-origin influenza A virus (S-OIV) H1N1 virus in humans. J Clin Virol 2009; 45(3):169-173.
PEIRIS M, Yuen KY, Leung CW et al. Human infection with influenza H9N2. Lancet 1999; 354(9182):916-917
QUAN FS, Compans RW, Nguyen HH, Kang SM. Induction of heterosubtypic immunity to influenza virus by intranasal immunization. J Virol 2008; 82(3):1350-1359.
RAGNI MV, Ruben FL, Winkelstein A, Spero JA, Bontempo FA, Lewis JH. Antibody responses to immunization of patients with hemophilia with and without evidence of human immunodeficiency virus (human T-lymphotropic virus type III) infection. *J Lab Clin Med* 1987; 109: 545–49.
RANIERI R, Veronelli A, Santambrogio C, Pontiroli AE. Impact of influenza vaccine on response to vaccination with pneumococcal vaccine in HIV patients. *AIDS Res Hum Retroviruse*s 2005; 21: 407–09.
REY D, Krantz V, Partisani M, et al. Increasing the number of hepatitis B vaccine injections augments anti-HBs response rate in HIV-infected patients. Effects on HIV-1 viral load. Vaccine 2000;18(13):1161-5.
RIMLAND D, Guest JL. Response to hepatitis A vaccine in HIV patients in the HAART era. AIDS 2005;19(15):1702-4
RUBEN FL, Jackson GG. A new subunit influenza vaccine: acceptability compared with standard vaccines and effect of dose on antigenicity. J Infect Dis 1972; 125(6):656-664
RUBEN FL, Potter CW, Stuart-Harris CH. Humoral and secretory antibody responses to immunization with low and high dosage split influenza virus vaccine. Arch Virol 1975; 47(2):157-166
SHIRE NJ, Welge JA, Sherman KE. Efficacy of inactivated hepatitis A vaccine in HIV-infected patients: a hierarchical bayesian meta-analysis. Vaccine 2006;24(3):272-9

STAPRANS SI, Hamilton BL, Follansbee SE, et al. Activation of virus replication after vaccination of HIV-1-infected individuals. *J Exp Med* 1995; 182: 1727–37.
SUBBARAO K, Klimov A, Katz J et al. Characterization of an avian influenza A (H5N1) virus isolated from a child with a fatal respiratory illness. Science 1998; 279(5349):393-396
SZRETTER KJ, Balish AL, Katz JM. Influenza: propagation, quantification, and storage. Curr Protoc Microbiol. 2006 Dec;Chapter 15:Unit 15G.1.
TASKER SA, Treanor JJ, Paxton WB, Wallace MR. Efficacy of influenza vaccination in HIV-infected persons: a randomized, double-blind, placebo-controlled trial. *Ann Intern Med* 1999; 131: 430–33.
TAYAL SC, Sankar KN. Impaired response to recombinant hepatitis B vaccine in asymptomatic HIV-infected individuals. AIDS 1994;8(4):558-9
TREANOR JJ, Campbell JD, Zangwill KM, Rowe T, Wolff M. Safety and immunogenicity of an inactivated subvirion influenza A (H5N1) vaccine. N Engl J Med 2006; 354(13):1343-1351
TREANOR JJ, Wilkinson BE, Masseoud F et al. Safety and immunogenicity of a recombinant hemagglutinin vaccine for H5 influenza in humans. Vaccine 2001; 19(13-14):1732-1737.
VASCONCELLOS MTL, Silva PLN, Szwarcwald CL. Sampling design for the *World Health Survey* in Brazil. Cad Saúde Pública 2005; 21 Suppl:S89-99
VESIKARI T, Pellegrini M, Karvonen A et al. Enhanced Immunogenicity of Seasonal Influenza Vaccines in Young Children Using MF59 Adjuvant. Pediatr Infect Dis J 2009; 28(7):563-571.
VIGANO A, Zuccotti GV, Pacei M et al. Humoral and cellular response to influenza vaccine in HIV-infected children with full viroimmunologic response to antiretroviral therapy. J Acquir Immune Defic Syndr 2008; 48(3):289-296.
WEINBERG A, Gona P, Nachman SA, et al. Antibody responses to hepatitis A virus vaccine in HIV-infected children with evidence of immunologic reconstitution while receiving highly active antiretroviral therapy. J Infect Dis 2006;193(2):302-11
WEISSMAN S, Feucht C, Moore BA. Response to hepatitis A vaccine in HIV-positive patients. J Viral Hepat 2006;13(2):81-6
WORLD Health Organization. http://www.carec.org/influenzaa-H1N1-pandemic-declaration.html. 2009
YAMANAKA H, Teruya K, Tanaka M, et al. Efficacy and immunologic responses to influenza vaccine in HIV-1-infected patients. *J Acquir Immune Defi c Syndr* 2005; 39: 167–73.
ZANETTI AR, Amendola A, Besana S, Boschini A, Tanzi E. Safety and immunogenicity of influenza vaccination in individuals infected with HIV. *Vaccine* 2002; 20 (suppl 5): B29–32.

Anexo 1 – Fluxograma de procedimentos do estudo – Parte 1

**Nome da consulta**	Triagem	Vacina H1N1	Telefônica	Telefônica	Vacinas H1N1 (HIV +) e sazonal (todos)	Telefônica	Telefônica	Pós-término de esquema vacinal completo (HIV+) e pós-influenza (todos)
**Dia de estudo**	- 20 a -1	Zero	1	7	21	7	27	42
**Janela**	Nenhuma	Nenhuma	Nenhuma	± 1 dia	0 a +4 dias	Nenhuma	± 1 dia	± 4 dias
Obtenção do TCLE	X							
História médica	X	X			X			X
Exame físico	X	X			X			X
Sinais vitais	X	X			X			X
Verificação de elegibilidade	X	X						
Alocação nos estratos de CD4 [1]		X						
Randomização [1]		X						
Vacina H1N1		X			X [1]			
Vacina influenza sazonal					X			
Reatogenicidade		X	X	X	X	X	X	
Fornecer diário do voluntário		X			X			
Recolher diário do voluntário					X			
Eventos adversos		X	X	X	X	X	X	X
Teste anti-HIV [2]	X							
Medicação concomitante	X	X			X			X
Exames de segurança [3]	X				X			X
Teste de gravidez [4]	X	X			X			
CD4 [1]	X				X			X
CV [1]		X			X			X
Sorologia H1N1		X			X			X
ELI-Spot		X			X			X

[1] Apenas para os voluntários com infecção pelo HIV

[2] Apenas para os que não conhecerem seu status sorológico previamente (candidatos ao grupo controle)

[3] exames de segurança são hemograma com plaquetas, TGO, TGP, bilirrubinas totais e frações

[4] Apenas com mulheres com potencial reprodutivo. Deve ser repetido a qualquer momento em que houver suspeita de gravidez

Anexo 1 – Fluxograma de procedimentos do estudo – Parte 2

**Nome da consulta**	8 semanas		16 semanas		24 semanas		32 semanas		40 semanas		48 semanas		Término
**Voluntários**	Todos	HIV+	Todos	HIV+	Todos	HIV+	Todos	HIV+	Todos	HIV+	Todos	HIV+	Todos
**Dia de estudo**	57	77	113	133	169	189	225	245	281	301	337	357	NA
**Janela**	± 7 dias		± 7 dias		± 7 dias		± 7 dias		± 7 dias		± 7 dias		± 7 dias
Obtenção do TCLE													
História médica	X		X		X		X		X		X		X [5]
Exame físico	X		X		X		X		X		X		X [5]
Sinais vitais	X		X		X		X		X		X		X [5]
Verificação de elegibilidade													
Alocação nos estratos de CD4 [1]													
Randomização [1]													
Vacina H1N1													
Vacina influenza sazonal													
Reatogenicidade													
Fornecer diário do voluntário													
Recolher diário do voluntário													
Eventos adversos	X		X		X		X		X		X		X [5]
Medicação concomitante	X		X		X		X		X		X		X [5]
Exames de segurança [2]													X [5]
Teste de gravidez [3]													
CD4 e CV [1]	X				X						X		X [5]
Sorologia H1N1	X		X		X		X		X		X		X [5]
ELI-Spot					X								X [5]

[1] Apenas para os voluntários com infecção pelo HIV

[2] Apenas para os que não conhecerem seu status sorológico previamente (candidatos ao grupo controle)

[3] exames de segurança são hemograma com plaquetas, TGO, TGP, bilirrubinas totais e frações

[4] Apenas com mulheres com potencial reprodutivo. Deve ser repetido a qualquer momento em que houver suspeita de gravidez

[5] Apenas se houve término precoce

NA = não se aplica

ANEXO V - ORÇAMENTO DO ESTUDO

Os pesquisadores não receberão nenhuma remuneração pelo desenvolvimento deste projeto de pesquisa. As atividades técnicas de acompanhamento clínico e de realização de exames laboratoriais serão realizadas pelas equipes de cada pesquisador / laboratório dentro de suas atividades rotineiras, sem pagamento adicional.

Reagentes e tubos (para sorologia, ELI-Spot e diagnóstico etiológico dos quadros respiratórios agudos): R$ 300.000,00.

Exames de segurança: R$ 54.000,00.

Reembolso de despesas com alimentação e transporte dos voluntários (450 voluntários, 16 consultas cada um, estimativa de R$ 20,00 de reembolso por consulta): R$ 144.000,00.

TOTAL: R$ 498.000,00

ANEXO VI - CRONOGRAMA DE EXECUÇÃO

	Março a Junho de 2010	Junho de 2010 a março de 2011	Abril a Junho de 2011
Inclusão de voluntários			
Acompanhamento dos voluntários			
Realização de sorologias e de ELI-Spot			
Análise final dos dados			
Publicação dos resultados			
